# Optimizing the design and implementation of question prompt lists to support person‐centred care: A scoping review

**DOI:** 10.1111/hex.13783

**Published:** 2023-05-25

**Authors:** Jessica U. Ramlakhan, Shazia Dhanani, Whitney B. Berta, Anna R. Gagliardi

**Affiliations:** ^1^ Toronto General Hospital Research Institute University Health Network Toronto Canada; ^2^ Institute of Health Policy, Management and Evaluation University of Toronto Toronto Canada

**Keywords:** patient–clinician communication, person‐centred care, question prompt list, scoping review

## Abstract

**Introduction:**

Question prompt lists (QPLs) are lists of questions that patients may want to discuss with clinicians. QPLs support person‐centred care and have been associated with many beneficial outcomes including improved patient question‐asking, and the amount and quality of the information provided by clinicians. The purpose of this study was to review published research on QPLs to explore how QPL design and implementation can be optimized.

**Methods:**

We performed a scoping review by searching MEDLINE, EMBASE, Scopus, CINAHL, Cochrane Library and Joanna Briggs Database from inception to 8 May 2022, for English language studies of any design that evaluated QPLs. We used summary statistics and text to report study characteristics, and QPL design and implementation.

**Results:**

We included 57 studies published from 1988 to 2022 by authors in 12 countries on a range of clinical topics. Of those, 56% provided the QPL, but few described how QPLs were developed. The number of questions varied widely (range 9–191). Most QPLs were single‐page handouts (44%) but others ranged from 2 to 33 pages. Most studies implemented a QPL alone with no other accompanying strategy; most often in a print format before consultations by mail (18%) or in the waiting room (66%). Both patients and clinicians identified numerous benefits to patients of QPLs (e.g., increased patient confidence to ask questions, and patient satisfaction with communication or care received; and reduced anxiety about health status or treatment). To support use, patients desired access to QPLs in advance of clinician visits, and clinicians desired information/training on how to use the QPL and answer questions. Most (88%) studies reported at least one beneficial impact of QPLs. This was true even for single‐page QPLs with few questions unaccompanied by other implementation strategies. Despite favourable views of QPLs, few studies assessed outcomes amongst clinicians.

**Conclusion:**

This review identified QPL characteristics and implementation strategies that may be associated with beneficial outcomes. Future research should confirm these findings via systematic review and explore the benefits of QPLs from the clinician's perspective.

**Patient/Public Contribution:**

Following this review, we used the findings to develop a QPL on hypertensive disorders of pregnancy and interviewed women and clinicians about QPL design including content, format, enablers and barriers of use, and potential outcomes including beneficial impacts and possible harms (will be published elsewhere).

## 1 INTRODUCTION

Patient–clinician communication is at the crux of high‐quality healthcare. Communication promotes a shared understanding of patient health beliefs and values, which directly and indirectly improves health outcomes.^1^ Communication establishes a healing and trusting patient–clinician relationship that helps patients feel supported, respected and empowered, which in turn can facilitate and improve medical decision‐making, enhance access to care, and improve treatment adherence, physiological processes and health, and psychosocial well‐being.^1^, ^2^ Patient–clinician communication is central to person‐centred care (PCC), referring to care that is tailored to individual needs, circumstances and preferences.^1^, ^3^ McCormack et al. conceptualized PCC using a rigorous three‐part methodology that engaged patients, family members, researchers and clinicians.^3^ This resulted in a comprehensive framework describing PCC across six domains: foster a healing relationship, recognize and respond to emotions, exchange information, address uncertainties and risks, support‐shared decisions and enable self‐management.^3^

Several systematic reviews have demonstrated the beneficial impact of PCC on patients, families, clinicians and health systems including the following: increased patient disease knowledge, satisfaction with consultations, treatment compliance and quality of life; decreased staff burnout and reduced hospital readmissions and improved cost‐effectiveness of healthcare.^4^, ^5^, ^6^ A 2018 meta‐analysis of 28 systematic reviews identified multiple strategies that support PCC categorized as communication tools, patient and/or communication skills training, health coaching, counselling, motivational interviewing, physical support, environmental changes and healthcare system processes.^7^ The meta‐analysis found that communication tools were the most frequently used strategy, leading to improved patient knowledge, self‐management of health, satisfaction with healthcare experiences and quality of life.^7^ While there is no widely‐accepted formal definition, in this context, ‘communication tools’ appear to be resources used by patients, family and/or clinicians to support person‐centred discussions during clinical consultations.^8^

Question prompt lists (QPLs) are an increasingly common tool to promote and support patient–clinician communication, typically consisting of a list of questions that patients may want to ask or discuss with clinicians.^9^, ^10^ While shared decision‐making is a component of PCC, QPLs can be distinguished from other communication tools such as decision aids, which help patients consider values and preferences to engage in shared‐decision making.^11^ QPLs enable patients to voice concerns and uncertainties via question‐asking, prompting patient–clinician discussion about any topic relevant to a particular condition or health concern of importance to patients, and not only decisions about treatment or management of a health issue.^9^, ^10^ QPLs have been used to support the discussion of various medical conditions amongst different populations. For example, an Internet search identified 173 QPLs on a wide range of topics.^12^ In general, encouraging patients to ask questions reduces their anxiety, and increases their knowledge and satisfaction with healthcare visits.^13^ QPLs appear to offer many benefits for patients, clinicians and health systems. For example, Brandes et al. conducted a systematic review of 16 studies on QPLs in the oncology setting and found QPLs enhanced patient question asking during consultation, satisfaction with consultation, knowledge and recall of information; and reduced anxiety at follow‐up.^9^ In another systematic review of 10 studies, cancer patients found QPLs more helpful than information sheets and may reduce anxiety at follow‐up visits.^14^ A rapid review of 42 studies on QPLs in health consultations by Sansoni et al. found that, in addition to the aforementioned benefits, QPL use increased the amount and quality of the information provided to patients by clinicians.^10^

No prior review characterized QPLs to identify common features, explore patient or clinician views on QPLs and examine other enablers or barriers of QPL use, or how QPLs were operationalized, knowledge needed to guide future development and implementation of QPLs. Lack of such knowledge impedes researchers and others from improving the design, implementation and evaluation of QPLs, limiting the potential of QPLs to support PCC. The purpose of this review was to synthesize published research that evaluated QPLs to explore how the design and implementation of QPLs may optimize QPL use and beneficial outcomes.

## 2 METHODS

### 2.1 Approach

We conducted a scoping review, which is a type of synthesis that examines the current state of research on a particular topic, particularly for topics that are relatively new, research is of mixed design, or concepts or design issues lack clarity, as is the case for QPLs.^15^, ^16^ We followed the methods initially proposed by Arksey and O'Malley, and refined by Levac et al.^15^, ^17^ We did not require institutional review board approval as data were publicly available, and we did not register a protocol because the International Prospective Register of Systematic Reviews does not accept scoping reviews. To optimize rigour, we adhered to the Preferred Reporting Items for Systematic Reviews and Meta‐analyses extension for scoping reviews.^18^

### 2.2 Scoping

We conducted a preliminary search to become familiar with the QPL literature. J. U. R. searched MEDLINE using select search terms employed by authors of prior QPL syntheses including question prompt and question asking.^9^, ^10^ J. U. R., W. B. B. and A. R. G. jointly reviewed search results to generate eligibility criteria and design a comprehensive search strategy.

### 2.3 Eligibility

We developed eligibility criteria based on the population, intervention, comparisons, outcomes and time framework,^19^ and refined those criteria prospective to screening. Supporting information: Additional File 1 offers detailed eligibility criteria that were needed to distinguish QPLs from a plethora of other types of tools for the purpose of screening. In brief, *population*s included patients aged 18+, family or caregivers or clinicians of any specialty who employed a QPL during a healthcare visit for any health issue in primary, secondary or tertiary healthcare settings in any country. *Interventions* involved the use of QPLs, characterized based on the Workgroup for Intervention Development and Evaluation Research (WIDER) recommendations for reporting behaviour change interventions, which we found was needed to understand how QPLs were operationalized to support decisions about inclusion or exclusion.^20^ This created a preliminary yet detailed QPL definition to facilitate screening (Supporting information: Additional File 1). We included studies that used various terms for QPLs including but not limited to: QPL, question prompt sheet, communication tool, communication aid, frequently asked questions list and commonly asked questions list. *Comparisons* referred to studies that evaluated single‐ or multifaceted interventions involving a QPL, where studies aimed to assess QPL use and/or impact alone or compared with other interventions, either before and after, or only after exposure to the QPL. Publication types included English‐language empirical studies based on qualitative, quantitative or multiple/mixed methods research design that evaluated a QPL. *Outcomes* included any reported by authors. No *time* restriction was applied; we included studies from database inception to the date of search. We excluded studies involving trainee or nonadult populations, and publications in the form of editorials, letters, opinions, protocols, abstracts or proceedings. We excluded studies of patient‐generated question lists alone, a very different type of intervention compared with preformed QPLs. We also excluded studies that developed a QPL but did not evaluate its use or impact. Reviews were not eligible but we screened reference lists for eligible primary studies.

### 2.4 Searching

We developed a comprehensive search strategy (Supporting information: Additional File 2) in conjunction with a medical librarian according to Peer Review of Electronic Search Strategy reporting guidelines.^21^ J. U. R. and S. D. searched MEDLINE, EMBASE, CINAHL, Scopus, Cochrane Library and Joanna Briggs Institute Database from inception to 8 May 2022. We did not search for the grey literature due to limitations noted by others: few repositories, no standardized methods for searching, resource intensive, low yield and potentially high risk of bias.^22^, ^23^

### 2.5 Screening

S. D., J. U. R. and A. R. G. independently screened titles/abstracts of the first 50 search results, and met to compare results, discuss discrepancies and refine eligibility criteria and the approach to screening. Thereafter, S. D. and J. U. R. independently screened the remaining titles/abstracts, periodically consulting with A. R. G. and W. B. B. to resolve uncertainties. S. D. and J. U. R. acquired and screened full‐text articles concurrent to data extraction.

### 2.6 Data extraction

We developed fields of data extraction based on those used in prior QPL reviews,^9^, ^10^ further elaborated by our study purpose, eligibility criteria and WIDER categories,^20^ and refined through the data extraction pilot‐testing process. Given little prior research that focussed on associating QPL characteristics with impact, we aimed to extract data on a wide range of characteristics. J. U. R., S. D. and A. R. G. independently extracted data from the same four studies, then met to compare and discuss findings, resolve discrepancies and refine the approach to data extraction. This process was repeated for the next six articles, at which time data extraction was congruent. Thereafter, J. U. R. and S. D. extracted data from all remaining studies, consulting with A. R. G. about uncertainties. Periodically, A. R. G. and W. B. B. reviewed data extraction to independently ensure accuracy, consistency and clarity. We extracted data on study characteristics (publication year, country, research design and healthcare issue), how the QPL was developed, QPL characteristics and how the QPL was implemented based on the WIDER reporting recommendations: participants, personnel and QPL content (e.g., clinical topic, questions, number of questions, number of pages), format (e.g., print or online, booklet or brochure), delivery (e.g., how QPL was shared),^20^ enablers and barriers to QPL use and impacts including the types of outcomes measured.

### 2.7 Data analysis

We used summary statistics, text and tables to describe the number of studies by publication year, country, research design and healthcare issue; QPL development processes and implementation strategies, and enablers, barriers and impacts of QPL use. The methodological quality of studies was not assessed as this is not required for scoping reviews.^15^, ^17^ We listed enablers and barriers of QPLs for patients and clinicians. To identify possible trends that could inform a future systematic review, we mapped QPL characteristics and implementation to impact, categorizing impact as either improved or no change. Impacts included any reported by studies such as the number of questions asked, satisfaction with communication or clinical outcomes.

### 2.8 Review by stakeholders

Methodologists advocate for engaging stakeholders at the results phase of scoping reviews.^17^ In line with this recommendation, we used the findings of this review about common and effective characteristics of QPLs to develop a QPL on hypertensive disorders of pregnancy and interviewed women and clinicians to refine QPL design including content and format (will be published elsewhere).

## 3 RESULTS

### 3.1 Search results

**Figure 1:**
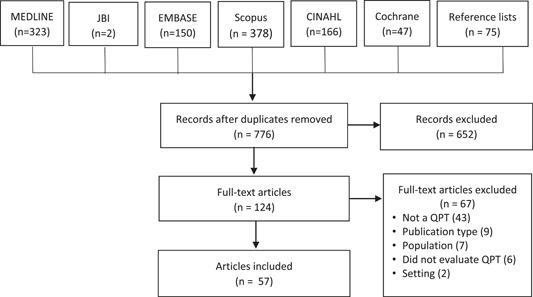
Preferred Reporting Items for Systematic Reviews and Meta‐analyses diagram.

The search generated 1141 results. We removed 386 duplicates and discarded 652 ineligible titles/abstracts. We excluded 67 full‐text studies because they did not investigate a QPL (*n* = 43), the population (*n* = 7), publication type (*n* = 9) and setting (n = 2) were ineligible, or did not evaluate a QPL (n = 6). Ultimately, we included 57 studies (Figure 1).

### 3.2 Study characteristics

Supporting Information: Additional File 3 provides data extracted from included studies.^24^, ^25^, ^26^, ^27^, ^28^, ^29^, ^30^, ^31^, ^32^, ^33^, ^34^, ^35^, ^36^, ^37^, ^38^, ^39^, ^40^, ^41^, ^42^, ^43^, ^44^, ^45^, ^46^, ^47^, ^48^, ^49^, ^50^, ^51^, ^52^, ^53^, ^54^, ^55^, ^56^, ^57^, ^58^, ^59^, ^60^, ^61^, ^62^, ^63^, ^64^, ^65^, ^66^, ^67^, ^68^, ^69^, ^70^, ^71^, ^72^, ^73^, ^74^, ^75^, ^76^, ^77^, ^78^, ^79^, ^80^ Studies were published from 1988 to 2022, with two‐thirds (38, 66.7%) published in the last 10 years. Studies were led by authors based in 12 countries, most often Australia (18, 31.6%) and the United States (17, 29.8%). Other countries included Germany, Korea, France, Sweden, The Netherlands, Italy, Norway, Japan and Singapore. Most QPLs were based on cancer topics (35, 61.4%). Two (3.5%) studies involved generic QPLs that were meant to prompt patients to ask questions about health topics of interest to them.^26^, ^35^ Other clinical topics, even for generic QPLs, included heart disease, reproductive health (polycystic ovarian syndrome, menopause), mental health (depression), asthma, arthritis, diabetes, HIV, neurologic health (migraine), dermatologic health (atopic dermatitis) and surgical care (pre‐ and postoperative procedures). Regarding research design, a large proportion of studies aimed to evaluate the impact of a QPL using a randomized controlled trial (31, 54.4%).

### 3.3 QPL development

About half of the studies provided the QPL either in the article or via an accessible Internet link (32, 56.1%). Most studies mentioned some details about how the QPL was developed (43, 75.4%), with many drawing from various sources to acquire content including pre‐existing QPLs, clinical guidelines, clinical consultation audio recordings or published research. However, in most studies, details about QPL development were vague or implied. For example, ‘The QPL group were shown a list of common questions (compiled by the researchers) which they could use for seeking clarification from their surgeons’.^62^

### 3.4 QPL design and implementation

#### 3.4.1 Content

Most often, QPLs included an introduction (17, 29.8%), instructions for use (28, 49.1%), blank space for noting answers or additional questions (28, 49.1%), section headings (25, 43.9%) and developer affiliations (6, 10.5%). For 51 studies in which it was reported, and excluding three‐question QPLs compared in three studies to lengthier QPLs,^26^, ^30^, ^31^ the number of questions included in QPLs varied widely (mean 51.0, median 39.0, range 9–191).

#### 3.4.2 Format

Amongst 32 studies in which it was reported, 14 (43.8%) QPLs were formatted as single‐page handouts, booklets or pamphlets, and 18 (56.3%) as multipage QPLs. Amongst multipage QPLs, the number of pages varied widely (mean 8.7, median 4.0, range 2–33).

#### 3.4.3 Delivery

Of the 57 studies, 2 (3.5%) did not provide QPL delivery details. Of the remaining 55 studies, 3 (5.5%) provided a print QPL to patients by healthcare professionals during consultations.^27^, ^42^, ^76^ Six (10.9%) studies provided the QPL to patients in electronic format before consultations by emailing or mailing them a link to a website.^25^, ^26^, ^35^, ^44^, ^66^, ^69^ Ten (18.2%) studies provided the QPL to patients in a print format before consultations by mailing it to them.^29^, ^38^, ^41^, ^45^, ^48^, ^70^, ^75^, ^77^ The remaining studies (36, 65.5%) provided a print QPL to patients in advance of consultations, either by a researcher (26, 72.2%) or a staff person or healthcare professional such as a social worker or nurse (10, 27.8%).

Nineteen (33.3%) studies evaluated a multifaceted intervention involving a QPL plus one or more additional strategies. Additional strategies most often were coaching on how to use the QPL (nine, 45.0%: six for patients, two for clinicians, one for both patients and clinicians),^24^, ^42^, ^48^, ^49^, ^52^, ^53^, ^57^, ^63^, ^76^ and educational material such as printed lay summaries (six, 30.0%).^59^, ^66^, ^67^, ^69^, ^71^, ^75^

### 3.5 Views about QPLs

A total of 26 (45.6%) studies assessed patient views about QPLs. Patients reported that QPLs were easy to use or understand,^26^, ^27^, ^33^, ^38^, ^39^, ^44^, ^47^, ^49^, ^54^, ^58^, ^71^, ^73^ and helped them in many ways: prepare for consultations with confidence,^25^, ^26^, ^34^, ^36^, ^38^, ^39^, ^44^, ^48^, ^58^, ^59^, ^65^, ^66^, ^73^ ask or discuss sensitive issues,^34^, ^38^, ^40^, ^45^, ^58^, ^64^, ^73^ communicate with clinicians,^27^, ^33^, ^34^, ^39^, ^46^, ^53^, ^58^, ^60^, ^63^, ^72^ identify or remember to ask questions,^35^, ^43^, ^56^, ^74^ prioritize questions,^24^, ^25^, ^26^, ^32^, ^39^, ^56^, ^60^ ask more questions than typical for them^24^, ^25^, ^27^, ^28^, ^33^, ^34^, ^38^, ^41^, ^45^, ^48^, ^50^, ^53^, ^54^, ^58^, ^59^, ^60^, ^61^, ^63^, ^64^, ^65^, ^66^, ^68^, ^69^, ^70^, ^71^, ^72^, ^73^, ^74^, ^75^, ^76^, ^77^, ^78^, ^79^, ^80^ and manage information overload.^34^, ^56^

In several studies, patients said they would recommend QPTs to future patients,^13^, ^37^, ^44^, ^45^, ^46^, ^54^, ^57^ and would use the QPT again.^38^, ^41^, ^42^, ^44^, ^45^, ^47^, ^52^, ^57^, ^58^, ^59^, ^74^

Fewer studies assessed clinician views about QPLs (17, 29.8%) and responses largely pertained to how QPLs helped patients rather than benefiting clinicians. Clinicians said that QPLs helped patients to communicate more easily, initiate discussions about sensitive issues, improve self‐efficacy around question asking, know how to frame thoughts into questions and reduce anxiety by equipping patients with knowledge.^25^, ^32^, ^34^, ^38^, ^39^, ^44^, ^46^, ^55^, ^57^, ^72^ Clinicians said that QPLs did not detract from clinical work;^55^, ^64^, ^65^ they would recommend QPLs to future patients and clinicians,^58^ and would use the QPL again.^72^

### 3.6 Enablers and barriers of QPL use

A total of 24 (42.1%) studies reported patient and/or clinician enablers or barriers to QPL use (Table 1). Enablers for patients included having sufficient time to read the QPL before appointments,^26^, ^43^, ^58^ translation to the patient's primary language and cultural acceptability of questions^43^, ^58^; not too many questions or too lengthy ones,^26^, ^31^, ^32^, ^48^ clinical contexts involving sensitive topics^34^, ^38^, ^52^, ^58^ and when physicians encouraged them to ask questions on the QPL.^26^, ^41^, ^43^ Patient‐reported barriers included the time required to read the QPL,^31^, ^58^, ^66^ being accompanied to a consultation,^28^, ^47^ if questions were addressed in some manner before QPL use,^58^, ^69^, ^76^ not having time to discuss QPL questions or additional questions during the consultation,^66^, ^76^ feeling overwhelmed,^33^, ^58^ feeling like clinicians disliked patient question‐asking,^76^ clinicians not being able to answer questions or discuss topics included in the QPL^24^, ^32^, ^33^, ^56^, ^72^ and QPLs not being available in a language other than English.^58^

**Table 1:** Enablers and barriers of QPL use reported by patients and clinicians.

Stakeholder	Enablers	Barriers
Patients	Good translation and acceptability of questions^43^, ^58^ Clinical contexts where QPLs support discussion of sensitive topics^34^, ^38^, ^52^, ^58^ Having a sufficient amount of time to read the QPL before a consultation^43^, ^58^ QPLs are a sufficient length‐there are not too many questions^26^, ^31^, ^32^, ^48^ QPL provided in an accessible format^26^ QPLs added in appointment software systems, or sent via SMS or mail^26^, ^58^ High information needs^52^, ^55^, ^56^ Physician encouragement^26^, ^41^, ^64^	Time‐consuming to read^31^, ^58^, ^66^ Time constraints of consultation^76^ Being accompanied to consultations^14^, ^28^ Questions answered prior to QPL use^58^, ^69^, ^76^ Fear of not being able to discuss questions not provided on QPLs^66^, ^76^ Feeling overwhelmed^33^, ^58^ Perceiving that clinicians dislike question‐asking^76^ QPLs not available in languages other than English^58^ QPL questions can cause patient anxiety^32^, ^33^, ^72^
Clinicians	Reliable supply of QPLs or other resources^25^, ^58^ Reminders for administrative staff to provide QPLs to patients^58^ Consultations scheduled for longer time periods^25^ Training on using QPL^24^, ^32^ Clinicians provided with QPLs prior to the consultation^44^, ^58^ QPL is not too long^44^	Time constraints for consultations with patients^25^, ^32^, ^58^ Clinician beliefs on efficacy and relevance of QPLs^76^ Difficulty remembering to use QPL^58^ Difficulty answering certain questions on QPL^25^, ^56^ Concerns about potential negative impacts on patients^24^, ^32^, ^58^ Clinicians lacking knowledge relevant to QPL questions^24^, ^32^, ^67^ Questions on QPL suitable for health setting^32^

Abbreviation: QPL, question prompt list.

Enablers for clinicians included receiving the QPL before consultations,^44^, ^57^ reliable supply of QPLs,^25^, ^57^ training on how to use the QPL,^24^, ^32^ the QPL is not too lengthy,^44^ and reminders for administrative staff to provide QPLs to patients and/or clinicians.^57^ Clinician‐reported barriers included clinician beliefs about the efficacy and relevance of QPLs,^75^ time constraints for consultations with patients,^25^, ^32^, ^57^ remembering to use the QPL,^57^ lack of knowledge about or difficulty answering QPL questions^24^, ^25^, ^32^, ^55^, ^66^ and concerns about perceived negative impacts on patients (e.g., increased anxiety).^24^, ^32^, ^57^

### 3.7 QPL impacts

Table 2 summarizes the benefits and harms associated with QPLs. Of 12 identified benefits, the most common were increased patient confidence to raise questions or concerns (26, 45.6%),^24^, ^25^, ^26^, ^27^, ^28^, ^33^, ^34^, ^39^, ^41^, ^43^, ^45^, ^46^, ^48^, ^50^, ^56^, ^58^, ^59^, ^60^, ^61^, ^62^, ^63^, ^64^, ^66^, ^69^, ^70^, ^72^, ^73^, ^75^ increased patient satisfaction with communication or care received (11, 19.3%),^25^, ^26^, ^27^, ^34^, ^38^, ^46^, ^55^, ^65^, ^71^, ^76^, ^78^ and reduced patient anxiety about their health status or treatment (10, 17.5%).^34^, ^38^, ^43^, ^46^, ^55^, ^57^, ^61^, ^70^, ^72^, ^74^ Of the three harms identified, the most common was confusion or anxiety arising from question topics or wording (7, 12.3%).^24^, ^32^, ^33^, ^47^, ^58^, ^59^, ^72^

**Table 2:** Reported benefits and harms associated with QPLs.

Type of impact	Impact	References
Benefits	Improved patient–clinician communication	[27, 33, 37, 49, 58, 62, 71]
	Improved patient–clinician relationship	[38, 65]
	Increased patient confidence and ability to ask questions or articulate concerns, including for sensitive topics	[24, 25, 26, 27, 28, 33, 34, 39, 41, 43, 45, 46, 48, 50, 56, 58, 59, 60, 61, 62, 63, 64, 66, 69, 70, 72, 73, 75]
	Helped patients to make decisions regarding care/treatment options	[25, 26, 33, 39, 55]
	Increased patient satisfaction with communication or care received	[25, 26, 27, 34, 38, 46, 55, 65, 71, 76, 78]
	Helped patients to prepare for consultations by prioritizing their concerns	[25, 26, 37, 44, 69]
	Patients felt informed about their condition or treatment	[25, 33, 34, 37, 56, 60, 65, 69]
	Helped patients feel involved or be involved in their care	[25, 26, 65]
	Reduced patient anxiety about their condition or treatment	[34, 38, 43, 46, 55, 57, 61, 70, 72, 74]
	Decreased length of consultation	[34]
	Improved quality of life	[37]
	Improved self‐efficacy to manage condition and/or symptoms	[36, 45, 73]
Harms	Question topics or words may cause confusion or anxiety	[24, 32, 33, 47, 58, 59, 72]
	Information overload	[32, 33, 67]
	Patients may not be ready to discuss certain topics	[52, 64]

Abbreviation: QPL, question prompt list.

Table 3 summarizes QPL impacts by QPL design and implementation. Of 57 included studies, 50 (87.7%) reported at least one beneficial impact. Of 50 (87.7%) studies that reported the number of questions included in the QPL (range 9–191), even those with fewer than 20 questions achieved beneficial impacts.^29^, ^33^, ^34^, ^39^, ^51^, ^54^, ^55^, ^67^, ^71^, ^74^, ^76^, ^78^, ^79^ Amongst 32 (56.1%) studies that reported QPL length in the number of pages, 12 (85.7%) of 14 single‐page QPLs achieved beneficial impacts. Amongst 38 (66.7%) studies in which the intervention consisted solely of a QPL, 31 (81.6%) achieved beneficial impacts.

**Table 3:** QPL impact by QPL design and implementation.

Study	Questions (*n*)	Format		Implementation		Impact	
		One page	Multiple pages	QPL only	Multifaceted	Improved	No change
Hjelmfors et al.^24^	45	NR	NR	X		Question‐asking Decide which questions to ask	–
Tracy et al.^25^	22	NR	NR	X		Question‐asking Confidence to ask questions Patient satisfaction Decide which questions to ask Patient decision‐making Attention to patient priorities Use of clinician time	–
Tracy et al.^26^	191		X	X		Involved in decisions Confidence to ask questions Decide which questions to ask	–
Bouleuc et al.^27^	112		X	X		Patient satisfaction Number of questions asked Communication with physician	Anxiety Depression Quality of life Consultation length
Buizza et al.^28^	50	NR	NR	X	X	Number of questions asked	–
Kalbfell et al.^29^	11	X		X		–	Conflict in decision‐making
Mariano et al.^30^	30		X	X		–	Involved in decisions
Roe et al.^31^	36		X	X		–	Involved in decisions
Van der Steen et al.^32^	76		X	X		Decide which questions to ask	–
Jayasekera et al.^33^	9	X		X		Question‐asking Communication of questions Knowledge of health/treatment Conflict in decision‐making	Distress
Kim et al.^34^	14	NR	NR	X		Patient satisfaction Manage information overload Question‐asking Communication of questions Knowledge of health/treatment Remember questions Confidence to ask questions Comfort with sensitive topics	Quality of life Self‐management
Tracy et al.^35^	25	NR	NR	X		Reviewing questions	–
Zetzl et al.^36^	NR	NR	NR	X		Confidence to ask questions	–
Buizza et al.^37^	50	NR	NR	X		Patient satisfaction Reduced anxiety	–
Yeganeh et al.^38^	156		X	X		Confidence to ask questions Question‐asking Comfort with sensitive topics	–
Berger et al.^39^	16	X		X		Preparing for consultation Understand decisions Patient satisfaction Communication of questions Patient control of care Decide which questions to ask Quality of life	Patient satisfaction Anxiety Trust in clinicians Knowledge of health/treatment
Best et al.^40^	112	NR	NR	X		Comfort with sensitive topics	–
Amundsen et al.^41^	49		X	X	X	Question‐asking	Anxiety Depression Patient satisfaction Quality of life
Hjelmfors et al.^42^	45		X		X	Clinician satisfaction	–
Hyatt et al.^43^	77	X			X	Remember to ask questions	–
Jacobs et al.^44^	75	NR	NR	X		Preparing for consultation	–
Khan et al.^45^	169		X	X		Question‐asking Comfort with sensitive topics Confidence for self‐care Reduced anxiety	–
Arthur et al.^46^	25	X		X		Communication of questions Reduced anxiety Visit length	–
Bottacini et al.^47^	50		X	X		–	Question‐asking Patient satisfaction Anxiety
Eggly et al.^48^	43	NR	NR	X	X	Question‐asking Confidence to ask questions Patient decision‐making	Consultation length Trust in physician
Epstein et al.^49^	NR	NR	NR		X	Patient satisfaction	–
Rodenbach et al.^50^	33	NR	NR	X		Question‐asking	–
McLawhorn et al.^51^	15	X		X		Increased do not revive orders Hospital referral rate	–
Walczak et al.^52^	44		X		X	Intention to use QPT	–
Brandes et al.^53^	39	NR	NR		X	QPL use Patient satisfaction Question‐asking Communication of questions	–
Hamann et al.^54^	15	X		X		–	Patient satisfaction Patient decision‐making Consultation length Question‐asking Questions answered
Yeh et al.^55^	12	X		X		Reduced anxiety Patient satisfaction	–
Walczak et al.^56^	44		X	X		Remember to ask questions Patient satisfaction Managing information overload Deciding on questions to ask	–
Aranda et al.^57^	NR	NR	NR		X	Reduced symptoms	Patient satisfaction
Dimoska et al.^58^	48		X	X		Question‐asking Comfort with sensitive topics Confidence to ask questions Communication of questions	–
Langbecker et al.^59^	189		X	X	X	Question‐asking Confidence to ask questions	–
Shirai et al.^60^	53		X	X		Deciding on questions to ask Question‐asking Knowledge of health/treatment	Patient satisfaction Question‐asking Communication of questions
Smets et al.^61^	38	X		X		Question‐asking	Consultation length Patient satisfaction
Lim et al.^62^	NR	NR	NR	X		Reduced anxiety	–
van Weert et al.^63^	NR	NR	NR		X	Communication of questions Question‐asking	Consultation length
Hebert et al.^64^	25		X	X		Question‐asking Comfort with sensitive topics	–
Clayton et al.^65^	112		X	X		Question‐asking Confidence to ask questions	Anxiety Patient satisfaction
Hartmann et al.^66^	NR	NR	NR		X	Confidence to ask questions Question‐asking Knowledge of health/treatment Patient‐physician relationship Patient decision‐making	–
Ogawa et al.^67^	17	X		X	X	Patient satisfaction	–
Glynne‐Jones et al.^68^	22	X			X	Patient satisfaction Question‐asking	–
Sciamanna et al.^69^	NR	NR	NR		X	Question‐asking	Patient satisfaction
Bolman et al.^70^	49	NR	NR	X		Receive more information Question‐asking Knowledge of health/treatment	Anxiety Patient decision‐making Patient satisfaction Consultation length
Butow et al.^71^	19	NR	NR		X	Question‐asking Patient anxiety	Patient decision‐making Consultation length Patient satisfaction Clinician satisfaction Clinician behaviour
Bruera et al.^72^	22	X		X		Communication of questions Patient satisfaction Question‐asking	Clinician satisfaction Consultation length
Clayton et al.^73^	114	NR	NR	X		Question‐asking Confidence to ask questions Reduced anxiety Comfort with sensitive topics	–
Brown et al.^74^	17	X		X	X	Question‐asking Reduced anxiety Remember to ask questions	Patient satisfaction
Martinali et al.^75^	49	NR	NR	X	X	Question‐asking Reduced anxiety	Patient decision‐making Patient satisfaction Knowledge of health or treatment Consultation length
Brown et al.^76^	17	NR	NR	X	X	Question‐asking	Anxiety Patient satisfaction Psychological adjustment to cancer
Fleissig et al.^77^	23		X	X		Prepare for consultations Question‐asking Patient satisfaction	–
Butow et al.^78^	11	X		X		Question asking	Patient satisfaction Psychological adjustment to cancer Patient recall of information
Thompson et al.^79^	13	NR	NR	X		–	Anxiety Patient satisfaction Question‐asking
Tabak^80^	33	NR	NR	X		–	Question‐asking

Abbreviation: NR, not reported.

## 4 DISCUSSION

This scoping review of 57 studies that evaluated QPLs on a range of clinical topics explored factors that may influence QPL impact including QPL design and implementation. Most (87.7%) studies reported beneficial impacts associated with QPLs. Most often, QPLs increased patient confidence to ask questions and satisfaction with communication or care; and reduced their anxiety about health status or treatment. This was true even for single‐page QPLs with few questions unaccompanied by other implementation strategies.

These findings build on prior research based on QPLs. Earlier research on QPLs largely focussed on the oncology setting,^9^, ^10^ but this review identified QPLs on a wide range of clinical topics, which in combination with other research,^12^ shows that QPLs are relevant to patients with a wide range of health concerns. Prior reviews of QPLs, conducted before 2015, largely focussed on QPL effectiveness.^9^, ^10^ This review updated prior reviews with current research, and by employing a scoping review approach, and exploring QPL design and implementation, expanded our knowledge of how to potentially optimize QPL effectiveness. By describing their characteristics in detail, this review revealed what constitutes a QPL.^11^ By focussing specifically on QPLs, this review highlights an important approach for supporting PCC not considered in a prior review of strategies for preparing patients before clinical visits,^81^ or a prior review of reviews on strategies to support PCC that identified patient information, involvement in care and empowerment as important strategies but did not discuss how to operationalize them.^7^ Overall, this study solidified the important role that QPLs can play in person‐centred communication and offers guidance to those developing and implementing QPLs.

Analysis of the findings offers insight into considerations for future QPL development. For example, only half of the studies included the QPL, two‐thirds described implementation and most offered vague details of how they developed the QPL. All future QPL research should more consistently report QPL design and implementation based on reporting guidelines such as the WIDER criteria.^20^ Only then can QPL developers, implementers and users fully understand how to optimize QPL development and implementation. Due to the limited detail about how QPLs were developed in most studies, it is not clear if or how patients were involved in QPL planning. Patient engagement in healthcare planning and improvement is becoming a worldwide standard.^82^ Patient engagement in designing QPLs represents another approach that could optimize QPL design, use and impact so that they meet patient needs and preferences. It would also be imperative to involve clinicians in planning QPL design and implementation to ensure that questions are clinically relevant and to optimize QPL adoption into clinical routines. One of the included studies that used a two‐round Delphi survey to assess acceptability found that most of the questions they rated were deemed acceptable by 96 patients and 26 healthcare professionals.^24^ In other research studies focussed on clinical decision support systems, which represent possible disruptions to clinical routines similar to QPLs, involving clinicians in system design resulted in significant improvement in awareness and adoption of clinical practices recommended by the system.^83^

From the patient's perspective, QPLs should be made available in advance of clinical appointments to allow time to review the questions. A systematic review found that previsit planning techniques targeting patients such as educational websites, telephone calls or self‐assessment tools improved patient–provider communication, illness perception and knowledge, perceived involvement in care and patient satisfaction with consultations.^81^ Patients also expressed concern that clinicians would not be open to using the QPL. A possible implementation strategy to address this risk is to disseminate the QPL to both patients so that they can prepare for appointments, and to clinicians so that they are primed for its use. This strategy is supported by a systematic review that found that interventions to support patient–provider communication are more likely to be adopted and impactful if shared with both groups rather than only one group.^84^

From the perspective of clinicians, this study found they were most concerned about lacking the knowledge to answer QPL questions and desired training on how to use the QPL. Hence, sharing of QPLs with clinicians might be accompanied by or direct clinicians to educational materials including published articles or clinical guidelines, or a version of the QPL with answers to the questions plus links to additional information on the topic. Another option is for professional societies to endorse the QPL and make it available in conjunction with accredited continuing education. In some included studies, clinicians expressed concern that QPLs might cause anxiety amongst patients. In contrast, patients thought that QPLs would reduce anxiety about health status or treatment. Hence, the implementation of QPLs by clinicians could include evidence of the many benefits of QPLs including reduced patient anxiety amongst others. These ideas are suggestions to overcome barriers reported in the included studies, which must be investigated through future research, as insight into strategies to support clinician adoption of point‐of‐care communication or decision tools is lacking.^85^, ^86^, ^87^

This study also identified knowledge useful to ongoing research. For example, since patients valued knowing what to ask and thinking of new questions, which in turn reduced anxiety and improved self‐efficacy, these are measures that could be evaluated in future QPL research, given that most studies included in this review assessed a number of questions asked. However, further research might engage patients in prioritizing the measures most important to them in terms of QPL benefits. When asked, most clinicians reported the benefits of QPLs for patients. Future research should explore how to enhance the perceived benefits of QPLs for clinicians, and more comprehensively assess the perceived and actual benefits of QPLs amongst clinicians. Few studies purposefully explored the harms of QPL use, so this should be consistently assessed in future research. To confirm the findings of this scoping review, a future systematic review is warranted to more definitively associate QPL design and implementation with beneficial impacts.

This study featured several strengths. The use of a scoping review approach to integrate the results of studies with different research designs revealed insight into how to optimize QPL development and implementation.^16^ We employed rigorous scoping review methods^15^, ^17^ and complied with standards for the conduct and reporting of scoping reviews and search strategies.^20^, ^21^ Several limitations should also be noted. As is the case with all syntheses, our literature search strategy may not have identified all relevant studies. The decision to exclude the grey literature and the stringency of screening criteria may have excluded potentially useful studies. While not required of scoping reviews,^15^, ^17^ the lack of critical appraisal of included studies means that interpretation and application of the findings must be interpreted with caution.

## 5 CONCLUSION

The purpose of this scoping review was to identify how to optimize QPL design and implementation. We included 57 studies that evaluated QPLs on a wide range of healthcare topics published from 1988 to 2022 by authors in 12 countries. Most (88%) studies reported at least one beneficial impact, most commonly patient confidence to ask questions, patient satisfaction with communication or care received; and reduced anxiety amongst patients about health status or treatment. This was true even for single‐page QPLs of <20 questions that included the following components: a brief introduction, instructions for use, blank space for writing notes, section headings and developer affiliations; and for QPLs alone, unaccompanied by other implementation strategies, when shared in printed format with patients before consultations (mailed home or in the waiting room) by either researchers, staff or healthcare professionals (e.g., nurse, social worker). To support implementation, clinicians wanted guidance on how to use QPLs. Many studies offered limited detail of QPL development, characteristics and implementation; hence, future research should more consistently do so by employing intervention reporting standards. To confirm these findings, future research should more definitively confirm how QPL design and implementation are associated with QPL impact.

## AUTHOR CONTRIBUTIONS

Anna R. Gagliardi conceptualized the study. Jessica U. Ramlakhan, Whitney B. Berta and Anna R. Gagliardi planned the study. Jessica U. Ramlakhan and Anna R. Gagliardi coordinated the study. Shazia Dhanani, Jessica U. Ramlakhan and Anna R. Gagliardi collected data. Shazia Dhanani, Jessica U. Ramlakhan, Anna R. Gagliardi and Whitney B. Berta analyzed and interpreted data. Shazia Dhanani, Jessica U. Ramlakhan, Whitney B. Berta and Anna R. Gagliardi drafted, edited and approved the final manuscript.

## CONFLICT OF INTEREST STATEMENT

The authors declare no conflict of interest.

## Supporting information

Supporting information.

Supporting information.

Supporting information.

## Data Availability

All data are available in the manuscript and additional files.
