# Correction to ‘Optimizing the design and implementation of question prompt lists to support person‐centred care: A scoping review’

**DOI:** 10.1111/hex.13882

**Published:** 2023-09-28

Dhanani S, Ramlakhan JU, Berta WB, Gagliardi AR. *Health Expectations* 2023;26(4):1404‐1417. doi:10.1111/hex.13783

The study was based on data collected by Jessica Ramlakhan for an MSc thesis. Although others re‐analysed the data and finalized the manuscript, the University of Toronto stipulates that graduate students must be first authors. Therefore, Jessica Ramlakhan will be the first author, and Shazia Dhanani the second author.

We apologize for the error.

