# Supplementary material for: Optimizing the design and implementation of question prompt lists to support person‐centred care: A scoping review
Source: Health Expect. 2023 May 25;26(4):1404–17. doi: 10.1111/hex.13783 (PMC10349246; doi:10.1111/hex.13783)
Supplement: Supplementary file 1 — Supporting information. [file HEX-26--s002.docx]

Additional File 1. Eligibility Criteria

**Objective**

To conduct a scoping review of the published QPL literature to date to identify the characteristics (e.g., design, format, delivery) of effective QPLs across conditions. Themes emerging from the scoping review can be used in future research to inform the design of a prototype QPL for women with hypertensive disorders of pregnancy (HDP).

**Definitions and key references**

Question prompt lists (QPLs) are a type of communication tool involving a list of questions or topics that patients may want to ask or discuss with clinicians (91,92). Since there is no standard definition or criteria for what constitutes a QPL, the WIDER recommendations for reporting behaviour change interventions has been used to thoroughly describe a QPL in Table 1 below (93). To further explain what a QPL is ***not*** Table 2 provides a comparison between QPLs and other similar but distinct communication tools, by explaining what makes QPLs unique.

1. Brandes K, Linn AJ, Butow PN, van Weert JCM. The characteristics and effectiveness of Question Prompt List interventions in oncology: a systematic review of the literature: QPL interventions in oncology. Psycho-Oncology. 2015 Mar;24(3):245–52.
2. Sansoni JE, Grootemaat P, Duncan C. Question Prompt Lists in health consultations: A review. Patient Education and Counseling. 2015 Dec;98(12):1454–64.
3. Albrecht L, Archibald M, Arseneau D, Scott SD. Development of a checklist to assess the quality of reporting of knowledge translation interventions using the Workgroup for Intervention Development and Evaluation Research (WIDER) recommendations. Implementation Sci. 2013 Dec;8(1):52.

Table 1**.** QPL description based on WIDER criteria^3^

| Criteria | Description |
| --- | --- |
| Recipients | Designed for use by patients or family members, either themselves or in collaboration with a clinician |
| Personnel or setting | Used in any healthcare setting. Can be provided to recipients by clinicians, health services organizations (e.g., healthcare clinic waiting area), or obtained by recipients in print or electronically from developers or other agencies |
| Developers | Typically developed by academic researchers, but have also been developed by government, government agencies, professional societies, charitable or advocacy organizations, disease specific foundations, health services organizations |
| Purpose | To promote patient engagement, participation, activation, and/or collaboration between patients and clinicians during health consultations; to improve the transfer of information between patients and clinicians in a way that is focused on the patients’ needs, preferences, values, and circumstances; and to prompt patients to acquire more information from clinicians (often via clinicians answering patient questions), thus helping clinicians reduce patient uncertainties or concerns. |
| Content | Includes health related questions or topics to prompt discussion. These can be specific or general across conditions and/or populations. Questions or topics can be about biomedical, psychosocial or other aspects of the health condition. QPLs may or may not include:   - Instructions for use - Information pertaining to: health condition (e.g., symptoms, treatment, psychosocial aspects) or QPL use (e.g., description of a QPL, importance of asking questions) - Blank space for patients to record: answers, new questions, reflection on values, interpretation and/or prioritization of questions/topics |
| Format | These can be either print resources in the form of a handout, booklet, checklist, frequently asked questions list, pamphlet, or digital resources such as web-based or mobile applications. |
| Delivery | If not acquired by patients themselves, they might be distributed to patients by professional, charitable, or advocacy organizations, or given to patients by their clinician immediately before consultation, multiple days before consultation, only during consultation, or after the consultation in preparation for a follow-up. QPLs may or may not be endorsed by clinicians. |
| Intensity or Duration | Can vary in length (e.g., number of questions/topics), and can also have multiple sections or chapters depending on the purpose and length of the intervention. Number of pages also varies, while a one-page handout is common. As mentioned above, they can be administered, before, during, or after the consultation, and can also be used subsequently by patients as desired. |

**ELIGIBLE**

**Population**

- Male or female adult patients (18+) with any health condition or undergoing any type of health consultation (e.g., no health condition with annual visit to general practitioner)
- Male or female adult (18+) caregivers (e.g., family, friends)
- Practicing physicians of any specialty in primary, secondary or tertiary care; nurses, or nurse practitioners
- Studies can be from any country (i.e., even developing countries)

**Intervention**

- QPLs – a list of questions or topics that patients may want to ask or discuss with their clinician.
- Synonymous terms may include but are not limited to: question prompt list, question prompt list, question prompt sheet, communication tool, communication aid, frequently asked questions list, commonly asked questions list, communication intervention
- May also be referred to generally as a: handout, booklet, checklist, leaflet or pamphlet
- Intervention must target patient-physician communication, patient participation in health consultations, and exchange of information between patients and clinicians (e.g., physicians, nurses).

**Comparisons**

- Publication type includes English language primary studies of any design that evaluated a QPL (pilot, qualitative, quantitative etc.)
- Investigates a QPL alone (single-faceted) or in combination with one or more interventions (multi-faceted – e.g., coaching followed by a QPL)
- Evaluates a single or multi-faceted intervention involving a QPL after, before-after, or in comparison to another intervention

**Outcomes**

- Any outcomes reported by articles that evaluate a QPL including but not limited to:

Table 3. Possible outcomes associated with QPLs

| During Consultation | After Consultation | In General |
| --- | --- | --- |
| - Number of questions asked/ topics raised - Patient satisfaction (with consultation, information, physician etc.) - Patient knowledge - Patient and/or clinician communication - Consultation duration - Feedback on intervention: comfort, usefulness, acceptability, qualitative feedback - QPL endorsed by clinician | - Clinical outcomes - Cost-benefit analysis - Quality of life - Anxiety - Self-management of symptoms - Impact on clinic flow - Referrals to other departments - Hospital readmission rates - Information needs fulfillment - Confidence in patient-clinician relationship - Patient or clinician communication skills - Patient or clinician views about QPLs | - Barriers and facilitators to QPL use/implementation - Patient or clinician or stakeholder views about or experiences with QPLs |

**Time**

No date restriction was applied

**NOT ELIGIBLE**

**Population**

- Trainee or allied health professional participants
- Non-adult participants (i.e., under 18 years old)

**Intervention**

- Studies where frequently asked questions were recorded and answers were provided to peer support group members. It’s not a QPL because although it’s a list of questions, it contains answers too, and it’s being sent to patients after the fact and it’s not used during consultations it’s being used in a peer to peer support group, so it’s not facilitating patient-clinician communication it’s only providing information to patients within a peer group, after the group
- Studies were frequently asked questions recorded and answers were provided as an informational resource rather than to prompt question asking, and more to help clinicians structure conversation following this resource

**Comparisons**

- Publication type/study design
  - Non-empirical research studies
  - Publications in the form of: editorials, opinion articles, protocols, abstracts, proceedings, conceptual analyses, case studies, patient resources and reviews (reference list of reviews was manually searched for eligible studies)
  - Studies that develop and/or validate a QPL, but do not evaluate a QPL
  - Studies that develop a new version of an existing QPL (e.g., adapting existing QPL to a new setting/population) and do not evaluate it
  - Evaluates a QPL designed for use outside of the health consultation (e.g., QPL to help patients obtain information from a researcher before consenting to participate in a clinical trial)
- Outcomes
  - Studies that only report outcomes related to decision making – e.g., a shared decision making tool incorrectly labelled as a QPL that only includes questions about treatment options and only reports outcomes to do with decision confidence, certainty etc.
  - Doesn’t study a QPL but concludes that QPLs are needed
