# Supplementary material for: Optimizing the design and implementation of question prompt lists to support person‐centred care: A scoping review
Source: Health Expect. 2023 May 25;26(4):1404–17. doi: 10.1111/hex.13783 (PMC10349246; doi:10.1111/hex.13783)
Supplement: Supplementary file 2 — Supporting information. [file HEX-26--s001.docx]

Additional File 2. Search strategy

| **#** | **Search Statement** | **Results** |
| --- | --- | --- |
| 1 | Patient Participation/mt [Methods] | 2691 |
| 2 | Physician-Patient Relations/ | 74626 |
| 3 | Health Communication/mt [Methods] | 1200 |
| 4 | Patient Education as Topic/mt [Methods] | 18469 |
| 5 | 1 or 2 or 3 or 4 | 95322 |
| 6 | (question* adj5 tool*).mp. | 6737 |
| 7 | (question* adj5 sheet*).mp. | 509 |
| 8 | (question* adj5 list*).mp. | 3085 |
| 9 | (question* adj5 checklist*).mp. | 2505 |
| 10 | (frequent* asked adj5 question*).mp. | 888 |
| 11 | (common* asked adj5 question*).mp. | 290 |
| 12 | (prompt adj5 question*).mp. | 462 |
| 13 | (ask* adj5 question* adj5 tool*).mp. | 121 |
| 14 | (ask* adj5 question* adj5 list*).mp. | 236 |
| 15 | (ask* adj5 question* adj5 sheet*).mp. | 17 |
| 16 | (ask* adj5 question* adj5 checklist*).mp. | 36 |
| 17 | question prompt*.mp. | 201 |
| 18 | or/6-17 | 14213 |
| 19 | 5 and 18 | 337 |
| 20 | limit 19 to english language | 306 |
| 21 | limit 20 to (case reports or comment or editorial or interview or lecture or letter or news or practice guideline) | 4 |
| 22 | 20 not 21 | 302 |
