# Supplementary material for: Optimizing the design and implementation of question prompt lists to support person‐centred care: A scoping review
Source: Health Expect. 2023 May 25;26(4):1404–17. doi: 10.1111/hex.13783 (PMC10349246; doi:10.1111/hex.13783)
Supplement: Supplementary file 3 — Supporting information. [file HEX-26--s003.docx]

Additional File 3. Data extracted from included studies

| Study | Objective | Research design | Population | Intervention | Outcomes |
| --- | --- | --- | --- | --- | --- |
| Hjelmfors 2022 **(24)**  Sweden  Heart Failure (HF) and End-of-Life Care | To address experiences of health professionals using a HF-QPL and their views on the role of end-of-life communication in heart failure care | Inductive and descriptive qualitative design | Sample size: 15  Sex: Female (100%)  Age: Mean 40 (SD 12)  Education: 13 nurses (87%) and 2 physicians (13%)  Mean 9 years working with patients with HF | Number and Type: Multi- faceted  **Pre-formed QPL + communication course on HF**  Recipients: Patients receiving HF and end-of-life care  Personnel or Setting: Provided to patients by health professionals at consultations in HF clinics  Developers: study authors  Purpose: To assess the impact of communication of health professionals in HF and end-of life care as well as positive/negative outcomes of using a HF-QPL with patients and family members.  Content: Questions based on five topics: 1. HF and what to expect in the future 2. Help and support during deterioration 3. end-of-life care issues 4. Questions for the family members 5. Questions about pacemakers and defibrillators  Format: A4 booklet in print  Delivery: Health professionals are provided the HF-QPL prior to the consultation to reflect on three aspects: 1. their knowledge to be able to answer questions and discuss questions with patients 2. questions that are difficult to answer and 3. their role in communication for prognosis of end-of-life based on their profession. Health professionals provide patients and/or one family member with the HF-QPL at the beginning of the consultation, and provide them with time (not specified) to read the QPL and highlight questions.  Intensity/Duration: 45 questions, no page count provided  Copy of QPL: Not provided in current paper. | The HF-QPL is a Bridge in Communication:   - QPLs acted as a guide in the conversation - Helped patients ask important questions - Patient and family chose which questions to discuss   Challenges of the Question Prompt List:   - Professionals felt the HF-QPL was difficult to use if they were not prepared to use the tool - Professionals felt afraid of different patient emotions during conversation using the QPL - Difficult for patients to continue a conversation and feel comfortable if physician lacks knowledge - Many professionals lacked knowledge on questions about devices - Questions on end-of life were hard to discuss - Separate individual discussions should happen with family members and one with patients due to different information needs, communication preferences, and to remain focused on patients' needs - Professionals posed a concern giving the HF-QPL to patients described “too healthy” or at beginning of HF trajectory - Fear of patients’ reactions when they look at QPL content |
| Tracy M 2022 **(25)**  Australia  Practitioner Perceptions | To investigate the views and experiences of health professionals using patient-generated prompt lists QPLs, and on patient-question asking during consultations | Qualitative Study | Sample Size: 39  Sex: 20 Female and 19 Male  Age: not given  Expertise: 23 specialist general practitioners, 8 medical specialty, 5 surgical specialty, 1 Pediatrics, 1 Obstetrics/gynecology, 1 Psychiatry | Number and Type: 1-single-faceted   - **Pre-formed sample QPLs with questions taken from Question Builder-Healthdirect Australia**   Recipients: patients visiting a clinic for a specific health and concern/condition and specialist general practitioners at 4 clinics in New South Wales, Australia  Personnel or Setting: 4 clinics in New South Wales, Australia  Developers: MCT (moderator of the study) using the Question Builder tool on Healthdirect government website of Australia  Purpose: Improve the utilization and effectiveness of QPLs  Content: 2 lists for GPs have 22 “New Symptoms”, or 18 “Follow-up questions”, each with 4 and 2 prioritized questions. Other specialist lists have 38 “first visit” or 22 “Follow-up questions” with 8 and 4 prioritized questions.  Format: online list (Question Builder on Healthdirect Australia website)  Intensity and Duration: Online (choosing from a list of most frequently asked questions)  Delivery: online (Question Builder on Healthdirect Australia website) provided by researchers of the study, and mentions that patients came to consultations with list. Sample QPL lists are provided to clinicians that are interviewed in this study.  Copy of QPLs: No | - QPLs could encourage patient question-asking - Patients could be more empowered to ask questions - Time and resources impact health professionals’ perceptions on QPLs - Professionals with one-hour consultations felt that QPLs are not a concern to be used. - Increase in patient satisfaction - More attention geared towards patient priorities during the consultation - Allow patients to decide what they want to ask to physicians - Patients requiring more information may not be able to use QPLs - Can be used for ensuring patient safety (to ensure all important information has been provided) - Can help doctors use allotted time effectively to address patient needs - Questions on QPL can benefit patient decision-making |
| Tracy MC 2022 **(26)**  Australia  QPLs for patients | To evaluate the impact of two generic QPLs in accessing health information for patients, as well as experiences accessing health information | Longitudinal Qualitative Study | Sample Size: 155  Groups:   - Interview Participants: 31 - Online survey Participants: 124   Sex:   - Interview Participants: 15 male and 16 female - Online survey participants: 34 male and 94 female   Age:   - Interview Participants: Mean age 46.3 - Online Survey Participants: Mean age 46.7   Education:   - Interview Participants: University degree 62%, Diploma/certificate 19%, Trade apprenticeship 0%, High school certificate or leaving certificate 16%, School certificate or intermediate certificate 3% - Online survey Participants: University degree 65%, Diploma/certificate 23%, Trade apprenticeship 1%, High school certificate or leaving certificate 9%, School certificate or intermediate certificate 2% | Number and Type: 2-single components   - Pre-formed Questions Builder (QB) with a list of questions - Pre-formed ASK tool (3 Generic Questions)   Recipients: Individuals from the Australian population  Personnel or Setting: Participants emailed a link to Question Builder or ASK after first and second interview  Developers: QB is an adapted Australian version of the QB made by the United States Agency for Healthcare Research and Quality. The ASK tool is the generic form with three questions.  Purpose: To assess the impact on health information needs from the introduction of the two generic QPLs over 3 months.  Content:   - QB: Questions relating to tests, symptoms, follow-up, and different types of appointments, such as first visit/follow-up - ASK: 3 generic questions: “What are my options?”, “What are the possible benefits and harms of that process” and “How likely are each of the benefits and harms to happen to me?”   Format: Both QB and ASK are online format  Delivery: Distributed to participants through an email link after the first and second interview before a third interview  Intensity and Duration: QB includes 101 unique questions and 191 questions from five different types of appointments, making 4 sheets of paper identified in study results. ASK is three generic questions  Copy of QPLs: No | Benefits of QPLs reported:   - Access to QPLs normalizes asking questions to clinicians - Some participants mention that QPLs Increases confidence for asking questions (ASK) - Uncertainty regarding ASK - Participants acknowledge the need to ask questions and want information during visits - Participants feel like they are given a choice to make decisions on care options - Feeling of empowerment among users - Participants felt involved and informed in health decisions - Participants describe the QPL to be easy to use - Helped participants prepare for a consultation and help them decide what is important to know - Hard to decide which questions to ask   Improvement to QPLs:   - Instructions were not clear - QB having 191 questions is not feasible (too many questions) - QPLs being accessible in formats such as apps - QPLs should be added in appointment software systems, or links should be sent through SMS in preparation for consultations - Leaflets or posters in waiting rooms would encourage the use of QPLs - QPLs should be a part of standard consultations and participants feel more comfortable using QPLs with physician encouragement to use QPLs |
| Bouleuc 2021 **(27)**  France  Advanced cancer (palliative care) | To assess the impact of a QPL adapted to French language and culture on communication about end-of-life issues during consultations. | Randomized controlled trial | Sample size: 142 patients with advanced cancer  Age: Median 59.6  Sex: 76.8% female  Education: 43.4% university education  Group differences:  Group 1: QPL  Group 2: Usual care | Number and type: 1 single-component  Recipients: Advanced cancer patients and caregivers  Personnel or setting: Palliative care consultations in outpatient clinics  Developers: Originally developed by research group Walczak et al. 2013 in the US and Australia (55). Adapted and translated to French language and culture by previous research by this group, Fouquet et al. (not in manuscript) researchers involving clinicians, and patients.  Purpose: To support communication and question-asking about palliative care given the highly sensitive nature of these discussions  Content: Layperson title; **introduction** / **instructions for use**; Questions about palliative care team, symptoms, treatment, symptom management, lifestyle, quality of life, prognosis and end of life; provided in English and French  Format: A5 booklet, provided in print, 12-point black times new roman, black text, white background, questions in bullet list, titles italicized  Delivery: Provided by clinicians at the end of consultation, and instructed to bring it to their follow up appointment and select questions they would like to ask. During consultation, QPL was endorsed by clinicians (clinicians reminded the patient that they had given them a QPL last time, and if they prepared any questions to go through).  Intensity / duration: 16 pages, 112 questions, 11 **sections with headings**  Copy of QPL: Yes (Appendix A, page 341.e1) | Anxiety (Hamilton Depression and Anxiety Scale): No significant difference (QPL 11.3, control 11.1, p=0.77)  Depression (Hamilton Depression and Anxiety Scale): No significant difference (QPL 4.3, control 3.8, p=0.47)  Quality of life (McGill Quality of Life Questionnaire): No significant difference (QPL 3.9, control 3.3, p=0.22)  Patient Satisfaction (patient satisfaction with cancer care questionnaire): QPL group significantly higher (QPL 13.1, control 11.9, p=0.024)  Number of questions asked: significantly higher in QPL group (25.4) than control (18.2) (p=0.01)  Consultation duration: No significant difference between QPL (33 minutes) and control (27 minutes) (p=0.07)  Patients said the QPL was: helpful with communicating with the physician (80%) and easy to understand (90%). Most patients (67%) read the QPL. |
| Buizza 2021 **(28)**  Italy  Breast Cancer | Evaluate the impact of patient-question asking during consultations using a QPS or QL, with or without a companion | Multi-centred Randomized Control Trial | Sample Size: 324  Sex: Female (100%)  Age: 18-75  Groups: (After recording lost due to technical issues-308)   - QPS Intervention Group: 158 patents with 120 accompanied - QL control group: 150 patients with 109 accompanied   Education: Highest proportion with High School and Secondary School education   - QPS Accompanied (120): High School 44 (36.6%) and Secondary School 38 (31.6%) - QPS Unaccompanied (38): High School 18 (47.3%), Secondary School 9 (23.7%) - QL Accompanied (109): High School 41 (37.6%), Secondary School (32 (29.3%) - QL Unaccompanied (41): High School 21 (51.2%), Secondary School 7 (17.1%), and University 7 (17.1%) | Number and Type: 2-single and2 multi-faceted   - **Pre-formed QPS +Accompanied by a companion** - **Pre-formed QL +Accompanied by a companion** - **Pre-formed QPS alone (Unaccompanied)** - **Pre-formed QL alone (Unaccompanied)**   Recipients: breast cancer patients 18-75 years old, with a recent breast cancer diagnosis, from stage I to stage III.  Personnel or Setting: three oncological sites in northern Italy  Developers: No information on developers of QPS or QL  Purpose: To identify if a QPS results in an increase in questions asked by patients than a QL.  Content: Questions on symptoms, aetiology, prognosis, prevention, treatment, bureaucracy in both QPS and QL. The QL consists of a self-generated list of questions by patients  Intensity and Duration:   - QPS: 50 questions, no information on page count - QL: No information on number of questions and page count   Format: QPS: sheet (no information provided), QL: sheet (no information provided)  Delivery: Both QPS and QL provided as sheets; no in-depth information on electronic or paper delivery; pre-consultation interventions  Copy of QPS or QL: Not provided in article | - QL patients asked more questions than the QPS group (M= 15.9, SD= 12.4 vs M= 13.4, SD =9.0) for all topics except prevention - Consultation time is equivalent for all groups   QPS intervention:   - Patients using the QPS and who were unaccompanied asked more questions than accompanied patients - Patients that used the QL and who were unaccompanied asked more questions than those that were accompanied - Patients using the QL unaccompanied had a higher score in decision making (SDMQ-9) than accompanied=more involved in consultation |
| Kalbfell 2021 **(29)**  US  High-Risk Surgeries | Evaluate the QPL for postoperative complications after high-risk surgery for individuals 60 and older | Multisite Randomized Control Trial | Sample Size: 20  Sex: 13 Male (65%) and 7 Female (35%)  Age: Mean 71 (60-86 years old)  Education: Not indicated Groups:   - QPL intervention: 12 (60%) - Control: 8 (40%)   Surgeons enrolled: 40  Surgeon Specialty: perform high-risk surgeries in older adults | Number and Type: 1-single faceted  Recipients: Patients over 60 years with minimum of one chronic condition undergoing high-risk surgery due to an oncological or vascular condition and met with a surgeon enrolled in the study  Personnel or Setting: QPL sent to patients before consultations. Setting is 5 sites including University of Wisconsin Hospital and Clinics, The University of California, San Fransisco, Medical Center; Oregon Health & Science Centre, Rutgers University Hospital, and Brigham and Women’s Hospital.  Developers: Authors of current research study  Purpose: To improve discussions about treatment options, what to expect after operations, and complications that might occur  Content: treatment options, recovery expectations, and complication management, a Notes section with **blank space** and **instructions; Sections Headings Should I have surgery? What should I expect if everything goes well? & What happens if things go wrong after surgery?**  Format: Tri-fold brochure  Delivery: Sent to patients before their consultation with a surgeon  Intensity and Duration: 11 questions, 1 page  Copy of QPL: Yes. QPL in Appendix A. Supplementary Data-Multimedia Component 1 | - No difference in conflict reported in the QPL intervention and control groups - A small portion of participants reported conflicts with the care management team on complications and treatment - Most participants received the care they needed and appreciated the care received |
| Mariano 2021 **(30)**  US  Orthopaedic Surgery | To investigate if the QPL for orthopedics improves perceived patient involvement in care compared to ASKShareKnow (3 generic questions list) | Pragmatic Randomized Control Trial | Sample Size: 156  Groups:   - QPL Intervention:78 - 3-Question Group: 78   Sex: 42 women in QPL (54%) and 46 women in 3-Questions (59%)  Age: Mean  55 QPL (SD=20) and 55 3-Q (SD=16)  Education:   - QPL: High School graduate 19%, Bachelor’s degree 37%, Master’s/other graduate degree 21%, Doctorate 13%, Trade school 9%, Some High school 1% - 3-Questions: QPL: High School graduate 33%, Bachelor’s degree 32%, Master’s/other graduate degree 15%, Doctorate 14%, Trade school 3%, Some High school 3% | Number and Type: 2-single components   - Pre-formed QPL - ASKShareKnow handout with questions “What are my options?”, “What are the possible benefits and harms of those options?”, and “How likely are each of those benefits and harms to happen to me?”   Recipients: patients with common orthopedic conditions visiting an orthopedic surgery clinic and older than 18 years of age.  Personnel or setting: Outpatient orthopaedic surgery clinic of an academic medical centre  Developers: Authors of research study; Developed using by taking questions from previous QPLs in palliative care and oncology and hand surgery. QPL reviewed by research team, which were six orthopedic surgeons and a patient advisory panel consisting of patients that have had orthopedic surgery.  Purpose: To improve patient perceived involvement in care  Content: Questions regarding themes of diagnosis, treatment options, support and outcomes. QPL displayed sections of questions for each theme with individual **themes as section headings.**  Format: Word Document Handout  Delivery: Patients received QPL or ASK handouts before visiting the surgeon. Surgeons were not informed whether patients received the QPL or ASK handout.  Intensity and Duration: 30 questions, 2 pages  Copy of QPL: Yes. In Supplemental Digital Content 1 ([http://links.lww](http://links.lww/).com/CORR/A481) | No significant difference in Perceived Involvement of Care Score (PICS) between QPL and the 3-Questions group   - QPL: 8.32 (SD=2.32) - 3-Questions: 8.46 (SD=2.29) - P=0.71 |
| Roe 2021 **(31)**  US  Common Hand Conditions | Evaluate the increase in patient involvement in care for common hand conditions using a QPL compared to ASK (3 generic questions) | Pragmatic Randomized Control Trial | Sample Size: 126  Sex: 69 female participants (55%)  Age: 18 years and older; mean patient age is 49 (SD=18.2)  Groups:   - Control group (ASK): 63 - Intervention group (QPL): 63   Education:   - Control Group: Master’s 18 (28.6%), Bachelor’s 17 (27%), High School graduate (27%) - Intervention: Bachelor’s 23 (36.5%), Master’s 13 (20.6%), High school graduate 12 (19%) | Number and Type: 2-single component   - **Pre-formed QPL** - **ASK 3-generic question list**   Recipients: Individuals with common hand conditions  Personnel or Setting: patients referred to two hand surgeons at Stanford University institution (R.K. and J.Y.)  Developers: Authors of the research study; Developed QPL by sending a questionnaire to patient advisory board, hand therapists and hand surgeons, conducted interviews with patients and refined the QPL based on feedback. The QPL is also assessed with the System Usability Score.  Purpose: To evaluate if QPL for patients with common hand conditions improves their involvement in care  Format: pamphlet, print format  Content: questions specific to common hand conditions on diagnosis, treatment, support and outcomes (**sub headings** added); **blank space** for notes on the title page and **affiliation** (VOICES Health Policy Research Center) on first page  Intensity and Duration: 36 questions, pamphlet, 2 pages  Delivery: Patients given the handouts by research assistants 5 minutes prior to meeting the hand surgeon  Copy of the QPL: Yes (Appendix A; available on the Journal’s Web site at [www.jhandsurg.org](http://www.jhandsurg.org/) ) | - There is no significant difference in Perceived Involvement in Care Scale (PICS) scores between QPL and ASK group (7.5 and 8.3) - A QPL for patients with common hand conditions may not be more effective than 3 generic question list (ASK) - QPL requires more time to read than the 3 generic questions (length of QPL) |
| van der Steen 2021 **(32)**  Netherlands  Palliative Care for Dementia: Practitioner Perceptions | To evaluate the QPL on palliative care and end-of-life for dementia patients, and views of QPLs among health professionals that work with dementia patients | Mixed Methods Evaluation Study | Sample Size:66 practitioners  Sex: 73% Women  Age: not specified, but 21 years of experience (SD 11)  Education: 2 general-based practice practitioners, 46 elderly care physicians, 1 geriatrician, 1 geriatric nurse and 18 general practitioners (GPs)  Experience: 56% care for patients with dementia daily | Number and Type: 1-single-faceted   - **Pre-Formed QPL**   Recipients: residents and supervisors in elderly care medicine and general practice. These professions specialize in primary care for dementia  Personnel or Setting: Two academic training centers for primary and long-term care in Leiden and Nijmegen universities, Netherlands.  Developers: a multidisciplinary team with draft presented to two panels of older adults from study centers with experiences in processes of dementia.  Purpose: to address thoughts about end-of life, to help patients think about questions for health professionals, and encourage patient-physician conversations.  Content: Content based on a previous booklet (Australian QPL for persons with dementia and their family, and a Candian QPL for family of nursing home residents with dementia). Includes three parts-1. Questions about illness (dementia and changes in health & Care goals, palliative care and end-of life decisions) 2. Questions about treatment and choices (decisions about treatments and agreements, treatment and care for common problems, end-of-life choices for prolonging and shortening life & choice of location of care and change of environment) 3. Questions about and for the relative (Care for you as a relative & the dying phase and after death)  Format: in the form of a booklet with information sample questions; in print; 2 pages  Intensity and Duration: 76 questions in total, and 2-11 questions per topic  Delivery: Survey and QPL distributed on paper at educational centers or sent by post mail by researchers of the study. Provided 2 copies per person.  Copy of QPL: supplemental file 1 | - Physicians scored the QPL as acceptable (mean acceptability score 51) with SD 10 - Mean usefulness: 7.2 on a 1-10 scale - Content mostly appreciated (mean quality is 64 with SD 10) - Most appreciated topic: care for relatives - Least appreciated topic: introduction about illness and care - 64% of physicians thought the QPL was too long - 59% of physicians felt there was too much information on the QPL - 49% of participants believed that individuals in early stages of dementia would not be able to use the QPL alone - Most physicians (56%) expected the QPL to increase provision of care - 35% of physicians mention the need for training to answer QPL questions   Enhancing conversations through discussing difficult topics   - QPL encourages patients and families to consider different questions on dementia and care options - Physicians are provided with topics to discuss with patients with QPLs - Possibility for information overload, leading to confusion or fear - Concerns using certain terms such as “advanced care planning” on QPLs - Concerns using certain questions on topics with no exact answer (e.g., disease progression overtime, life expectancy and religion) as they cause patient anxiety.   End-of-life discussions:   - Education level: high patient education level= patients understand the condition and are able to contribute to conversation with the health professional - Relevance of setting where QPL conversation occurs: patients living in nursing home residents having the chance to think of end-of-life than individuals living in community settings.   QPL implementation:   - Some physicians want QPLs to be implemented soon - Lack of time to discuss many questions if there are many questions on QPL - QPLs formatted with many questions not suitable in health setting |
| Jayasekera 2020 **(33)**  US  21-Gene Recurrence Test for Breast Cancer | To evaluate the QPL’s feasibility, knowledge, conflict in decision making and distress on testing and treatment for women with early-stage breast cancer | Not Specified | Sample size: 201  Groups:   - MEND 1 (136): do not receive QPL to assess distress and conflicts regarding decision-making - MEND 2 (65): receive QPL to assess feasibility   Age:   - MEND 1: Mean 56.9 SD 9.9 (28-83) - MEND 2: Mean 60.2 SD 11.0 (35-77)   Education:   - MEND 1: 81 (59.6%) with college degree/graduate/professional training, 54 (39.7%) <High school degree/some college, 1 (0.7%) missing - MEND 2: 38 (58.5%) with college degree/graduate/professional training, 27 (41.5%) <High school degree/some college | Number and Type: 1-single faceted   - **Pre-formed QPL**   Recipients: MEND 2 women; women with early-stage breast cancer receiving 21-gene RS testing  Personnel & Setting: MEND 2 women mailed at the QPL to bring to next oncology appointment  Developers: Authors of research study; QPL made through literature review and interview with 10 patients and 5 medical oncologists.  Purpose: To provide guidance in decision-making for treatments and support to women receiving 21-gene RS testing  Content:   - RS: impact on treatment and information on RS - Hormonal and chemotherapy as ways to reduce breast cancer from occurring another time - How it is important to follow all precautions and instructions for hormonal therapy - Adverse effects of hormonal therapy - **Blank space** for patients to write their own questions and key terms - Includes **affiliation** on first page   Format: Mailed to patients and they had to bring a copy to the consultation. They were provided with another copy if they did not bring one.  Delivery: MEND 2 patients mailed, and asked to bring a copy of QPL to the next appointment to discuss 21-gene recurrence score and treatment.  Intensity and Duration: 9 Questions, one-page with four sections **(section headings added)**  Copy of QPL: Yes, in Appendix 1 of article (Figure A1, page e1094) | Feasibility and Acceptability:   - 98.3% of participants in MEND 2 found QPL easy to understand - 71.2% reported the QPL helped ask questions to oncologists - 82% found the QPL helpful - 83.3% report the QPL allows patient to ask questions easily - 81.4% report the QPL to communicate questions they have - 11.7% report QPL giving anxiety, and was overwhelming to read - 40% report asking more questions with QPL   Knowledge and Distress   - Increase in knowledge on treatment and overall knowledge after using QPL - No change in knowledge on 21-gene RS testing and distress   MEND 1 and MEND 2 Distress   - No statistical difference found in distress among MEND 1 (no QPL) and MEND 2 (QPL) - MEND 2 women had lower conflicts regarding decision-making |
| Kim 2020 **(34)**  Republic of Korea  HIV | To pilot test the feasibility of a QPL for patients with HIV. | Questionnaire | Sample size: 18 patients with HIV  Age: Mean 34.9 (SD 12.34)  Sex: 5.6% female  Education: NR  Group differences: N/A | Number and type: 1 single-faceted   - **Pre-formed QPL**   Recipients: Patients newly diagnosed with HIV  Personnel or setting: Outpatient clinics for HIV  Developers: Researchers with physicians, nurses, and clinical psychologists  Purpose: To help patients and clinicians discuss a new HIV diagnosis, given the sensitive nature of this topic.  Content: Questions that patients can ask their clinicians about a new HIV diagnosis from the standard counseling manual for HIV. Details NR.  Format: In print, details NR  Delivery: Clinicians provided the QPL to patients in the waiting room before consultation. The clinician provided verbal instruction to check off any questions they wanted to ask.  Intensity / duration: 14 questions, pages NR  Copy of QPL: Not provided in current paper. (Not in manuscript) | Patient satisfaction: Most patients were satisfied with the intervention (self-report 6 item survey, Mean score: 7.3 / 10)  Communication with healthcare provider: No significant difference (29.3 pre-QPL versus 29.6 post-QPL, p=0.799)  Health related quality of life: No significant difference (69.4 pre-QPL versus 74.4 post-QPL, p=0.74)  Self-management: No significant difference (46.2 pre-QPL versus 48.1 post-QPL, p=0.3)  Patients thought QPL was helpful (16 of 18 gave positive answers; 3 of 18 provided suggestions for improvements):   - Manages information overload - Asking questions that they normally wouldn’t have - Put concerns into words - Increase knowledge about HIV - Prepare for consultation - Decrease consultation length / make use of short time - Helped to remember questions   Clinician views about QPL:   - Helped patients communicate about sensitive issues (e.g., sexual activity) - Feel confident asking questions, especially in busy outpatient setting - Helped patients frame thoughts into questions - Reduces patient anxiety by giving them knowledge |
| Tracy 2020 **(35)**  Australia  Generic (any condition) | To evaluate the uptake of a QPL that was passively promoted on a government consumer health information website. | Qualitative content analysis of user data | Sample size: 107 users (patients with heterogeneous conditions)  Age: NR  Sex: NR  Education: NR  Group differences: N/A | Number and type: 1 single-faceted   - **Pre-formed QPL**   Recipients: Any patient who accesses the online QPL  Personnel or setting: QPL is publically available on the HealthDirect Australia health consumer website. This website is typically used by 3.5 million people every month.  Developers: Developed in 2007 by the United States Agency for Healthcare Research and Quality as part of a national campaign called “Questions are the answer.” Healthdirect Australia was engaged by the Australian Commission on Safety and Quality in Health Care to adapt the QB-USA for Australia. Development methods NR, associated publications NR  Purpose: To encourage patient question asking during consultations with healthcare providers.  Content: Customized QPL based on clinical context. Users access the QPL via the government HealthDirect website and then select who their next appointment is with (family physician or specialist), what type of consultation it is (first appointment, follow up), and then a list of generic questions appear relevant to tests, treatment, medications, self-management, and follow up. Then, patients are provided with a list of questions and they can select, prioritize, and print / email their question list. They can also view a list of questions that their doctor may ask them for which they may want to prepare answers.  Format: Web-based.  Delivery: N/A – patients access the website passively and decide how to use QPL  Intensity / duration: Total 101 questions. Question options presented to patients, mean 25 questions depending on consultation type  Copy of QPL: Yes: <https://www.healthdirect.gov.au/question-builder> | QPL Use:   - Accessed 8915 times - 4000 users started creating QPLs - 2444 QPLs built for general consultations - 1556 created for specialist consultations - 40% of total questions were about shared decision making - Most frequently chosen question was “do I need any tests?” |
| Zetzl 2020 **(36)**  Germany  Heterogeneous Cancer | To assess the impact of a QPL on communication about radiotherapy.  . | Randomized controlled trial | Sample size: 279 patients with cancer undergoing radiotherapy  Age: Mean 64.9 (SD: 11.9)  Sex: 46.2% female  Education: low (53%), intermediate (23.3%), high (19.0%)  Group differences:  Group 1: QPL  Group 2: Usual care | Number and type: 1 single-faceted   - **Pre-formed QPL**   Recipients: patients with heterogeneous cancers  Personnel or setting: radiation oncology outpatient clinic  Developers: NR  Purpose: To support exchange of information and help with implicit expression of emotions  Content: Questions about physical complaints, need for information on support and palliative care. **Blank space** to add more questions.  Format: In print  Delivery: Provided to patients before consultation in the waiting room. Who delivered by NR.  Intensity / duration: NR  Copy of QPL: Not provided | Self-efficacy: Significant increase in interactional empowerment (interactional empowerment questionnaire iE-Q) in QPL group versus control (p=0.007)  QPL Use: 60.4% of participants in QPL group reported using the QPL with medical team |
| Buizza 2020  **(37)**  Italy  Breast Cancer | To compare the impact of a pre-formed QPL versus patient-generated QPL | Randomized control trial | Sample size: 324 women with early-stage breast cancer; 20 oncologists  Age: Mean 56 (patients); Mean 38.8 (clinicians)  Sex: 100% female (patients); 85% female (clinicians)  Education: 18.4% primary school, 27.9% secondary, 41.3% high school, 12.45 university (patients); mean 11.3 years of experience as medical oncologist (clinicians)  Group differences:  Group 1: Patient-generated QPL  Group 2: Pre-formed QPL and patient-generated QPL | Number and type: 2 single-component interventions   - **Compared Patient-generated and Pre-formed QPL**   Recipients: *Patient-generated and pre-formed QPL* Patients with early-stage breast cancer  Personnel or setting: *Patient-generated and pre-formed QPL* Three hospital-based outpatient oncology clinics  Developers: *Patient-generated and pre-formed QPL* Previously developed by this group of researchers based on studies in the field (not in manuscript)  Purpose: *Patient-generated and pre-formed QPL* Originally designed to prompt patients to consider novel topics and ask questions (not in manuscript)  Content: *Patient-generated QPL* Instructions for use (participants are asked to write a list of questions they would like to ask their oncologist) *Pre-formed QPL* Referred reader to protocol (not in manuscript)  Format: *Patient-generated and pre-formed QPL* Printed copies given in clinic (no further details reported - referred reader to protocol (not in manuscript)  Delivery: *Patient-generated QPL:* Participants were asked to write a list of questions they would like to ask their oncologist *Pre-formed QPL:* Referred reader to protocol (not in manuscript)  Intensity/Duration: *patient-generated QPL:* one-page blank sheet *pre-formed QPL*: 50 questions  Pre-formed QPL intervention details: See protocol (not in manuscript) | Oncologist perception of patient difficulty:   - Patients generally not perceived as difficult by oncologist: Difficult Doctor-Patient Relationship (30 or greater indicates difficult patient) mean (SD) pre-formed QPL 22.99 (8.7), patient-generated QPL 22.43(8.7), p=0.57 - High oncologist perceived difficulty weakly correlated with low patient satisfaction with oncologist relationship: r=-0.135, p=0.033   Patient satisfaction with oncologist relationship: High across groups, Patient-Doctor Relationship Questionnaire Mean scores: QPL 42.67 (4.1) vs. PGQPL 42.64 (4.2), p=0.95  Anxiety: Decreased post-consultation across groups. State-Trait Anxiety Inventory pre-consultation mean scores: QPL 47.32 (11.2) vs. PGQPL 48.83 (12.4), p=0.27; State-Trait Anxiety Inventory post-consultation: QPL 19.52 (6.3) vs. PGQPL 19.28 (6.3), p=0.75 |
| Yeganeh 2020 **(38)**  Australia  Early Menopause | To develop and pilot test a QPL for early menopause. | Multiple methods: survey and qualitative interviews | Sample size: *Interviews round 1*: 18 women with early menopause; *Interviews round 2:* 11 women with early menopause and 6 clinicians  Age:  *Interviews round 1:* Mean 52.41 (SD: 11.42) *Interviews round 2:* patients: 38.64 (SD: 10.26); clinicians: NR  Sex: *Interviews round 1:* 100% female  *Interviews round 2:* Patients: 100% female; clinicians: NR  Education: *Interviews round 1:* 82.3% Undergraduate or less *Interviews round 2:* Patients: 54.5% high school or less; Clinicians: 4 gynecologists, 2 endocrinologists  Group differences:  *Interviews round 1:* Interviews with patients only to determine acceptability before pilot testing; *Interviews round 2:* Interviews with patients and clinicians after using QPL to pilot test feasibility | Number and type: 1 single-faceted   - **Pre-formed QPL**   Recipients: women with early menopause  Personnel or setting: outpatient clinic for menopause  Developers: Researchers with women with early menopause, and clinicians (family physicians, endocrinologists, gynaecologists) via a survey and qualitative interviews  Purpose: Facilitate communication about early menopause given the sensitive nature of these discussions / this topic.  Content: title, **instructions for use**, pre-formed list of questions, links to online information, table of contents, and **section headings** (e.g., Causes, diagnosis, symptoms, Long-term effects of early menopause, treatment of early menopause, Support groups and where to seek help & Additional Questions)  Format: Available electronically via a website and in print. Questions presented in bullet list, purple and turquoise titles, black text. Bold to emphasize titles. White background. Booklet. Graphics (picture of words relevant to early menopause)  Delivery: Mailed a paper copy of the QPL a week before consultation. Researchers instructed women to read the QPL and use it during consultation if they wished.  Intensity / duration: 19 pages, 2 sections, 156 questions  Copy of QPL: Yes: <https://www.healthtalkaustralia.org/wp-content/uploads/2019/12/QPL-V3-Dec-2019.pdf> | Patient views on QPL:   - Patients said that the QPL was comprehensive, user friendly, informative and empowering - 100% perceived QPL as helpful - 81.8% would use QPL again - Most women asked 1 to 2 questions (73%)   Clinician views on QPLs:   - Helped patients ask questions - Helped patients initiate discussions about important and sensitive issues |
| Berger 2019 **(39)**  US  Outpatient Cancer Care (Heterogenous Cancers) | To evaluate the impact of the NCCS tool (QPL) on patients receiving cancer care and their health professionals | Before-and-after pilot study | Sample Size: 15 clinicians and 90 patients  Sex (Patients): 39 female (43%)  Sex (Clinicians): 5 females  Age (Patients):   - Pre-Intervention Group: Mean 63.1 (SD= 11.49) - Post-Intervention Group: Mean 62.75 (SD=10.47)   Education (Patients):   - Pre-Intervention Group: 36.7% post-graduate work, 23.3% college degree, 23.3% some college, 16.7% High school graduate - Post-Intervention Group: 43.3% post graduate work, 30% college degree, 15% some college, 10% high school graduate   Groups:   - Pre-intervention: NO NCCS tool - Post-Intervention: NCCS tool provided   Clinician Expertise: a mean 14 years of practice | Number and Type: 1-single Faceted   - **Pre-Formed QPL (NCCS Know Yourself Tool)**   Recipients: patients with lung, breast, genitourinary, head and neck cancer and gastrointestinal cancer  Personnel or Setting: Research assistant provides the form to patients before the consultation with the specialist  Developers: The National Coalition for Cancer Survivorship. Developed from analyzing the “Smith Form” used for decision making  Purpose: to identify feasibility and use of QPL among cancer patients and specialists  Content:   - Title: Take Charge of Your Cancer Care- Know Yourself - **Introduction:** Two sentences - Discussion Questions on treatment, cost, information about condition, cure and services - Additional Questions section that may or may not be applicable to patients: **blank space** for patients to write any additional questions, and questions on time remaining, guardians, legal affairs, and setting of care - Includes **affiliation** at the top of the page (NCCS)   Format: in print; it is designed to be printed  Intensity and Duration: 12 Discussion Questions, 4 Additional questions that may or may not be applicable and blank space for additional questions. The NCCS Tool is one page long.  Delivery: Patients receive the form and instructions to use the form verbally in-person from a research assistant before the consultation.  Copy of QPL: Yes, in the article (Figure 1, page 3) | Participants:   - 49% of patients that use the NCCS tool report helps prepare for consultations and understand their reasoning for decision-making - 91% report the tool is easy to use - 73% would recommend the tool to others - 47% of patients felt the tool improves quality of care - 56% of patients report the tool made communication with clinicians less difficult - 55% report having control of care using the tool - 94% believe the length is good for the tool   Clinicians:   - 74% report the tool being helpful to consider concerns - 67% report the tool to facilitate the patient-clinician interaction - 62% report tool improves communication - 70% report tool helps to understand needs of patients - 71% report the tool to increase Quality of Life   No significant findings were reported for patient satisfaction (PSQ-18), anxiety (GAD-7) and patient trust in clinicians, and uncertainty of care from clinicians between pre-intervention and post-intervention groups. |
| Best 2019 **(40)**  Australia  Advanced cancer (palliative care) | To evaluate the impact of a QPL on spirituality discussions in palliative care consultation | Randomized controlled trial | Sample size: 174 advanced cancer patients  Age: Mean 65, SD 13.3  Sex: 39.5% female  Education: 10 ≥ years 67.2%; 12 > years 13.2%; tertiary education (university and non- university) 19.5%  Group differences: Group 1: QPL  Group 2: Usual care | Number and type: One single-component   - **Pre-formed QPL**   Recipients: Advanced cancer patients and caregivers  Personnel or setting: Palliative care consultations in hospitals, inpatient units, and community settings (98% were in outpatient clinics)  Developers: This research group, in a previous study (63). QPL was previously evaluated in a randomized controlled trial, and shown to significantly increase question asking and discussion about prognosis and end-of-life care, without creating patient anxiety or impairing satisfaction.  Purpose: To influence patient and caregiver questions and discussion of topics relevant to end-of-life care during consultations with a palliative care physician. Not specifically designed to influence spirituality discussions  Content: Multiple topics and questions relevant to end-of-life care, including 4 questions about spirituality (related to faith, peace, and finding meaning in life)  Format: print  Delivery: Provided to patients 20-30 minutes by researcher prior to either their first, second, or third consultation.  Intensity/duration: NR  Copy of QPL: Developed and evaluated in parent study (65) | The following were hand-coded from consultation audio-recording  Spirituality discussion: Non-significant increase in discussion of spirituality in QPL group vs. control; 58.8% vs. 45.6%, p=0.295 |
| Amundsen 2018 **(41)**  Norway  Heterogeneous Cancer | To investigate the impact of a QPL alone and in combination with a consultation audio recording | Non-randomized controlled trial | Sample size: 93 cancer patients; 22 oncology physicians  Age: Mean 60 (patients)  Sex: 50.5% female (patients); 44% female (physicians)  Education: 41.9% > 10 years; 38.7% = 10 years; 19.4% < 10 years (patients)  Group Differences  Group 1: QPL only  Group 2: QPL + consultation audio recording  Group 3: Control (regular care) | Number and type: 1 single-component and 1 multifaceted   - Pre-formed QPL alone - Pre-formed QPL + consultation audio recording (provided to patient on a memory stick immediately after consultation)   **Pre-formed QPL Description**  Recipients: Patients and physicians  Personnel or setting: Hospital-based city-based outpatient cancer clinic  Developers: Previous researchers in Australia. Adapted to Norwegian context by this group; validated and shown to have high patient acceptability in previous research (41)  Purpose: To encourage targeted question asking  Content: **Instructions for use;** questions generally applicable to oncology consultations; and a **blank page** to write any additional questions  Format: Printed A5 booklet  Delivery: Mailed to patients prior to consultation  Intensity/Duration: 4 pages, 14 categories, 49 questions  Copy of QPL: See (41) Appendix A | The following were derived from consultation audio-recordings  Number of questions asked: No group differences. Both QPL groups (analyzed together for this measure) Mean (SD) 23(17), Control 17(15), p=0.070  Physician endorsement: Across both QPL groups, physician endorsement of QPL early in consultation significantly increased number of questions asked compared to endorsement later in consultation early mean 35 questions; late: mean 19 questions; p=.008  Content of questions asked: Across both QPL groups, participants asked more questions about prognosis, disease, quality of treatment, compared to control. Prognosis: both QPL groups 1.7, control 0.1, p<0.001; Disease: Both QPL groups: 5.0, control 2.5, p=0.001; Quality of treatment: Both QPL groups: 1.5, control 0.1, p<0.001  QPL Use (self-reported in post-consultation questionnaire, both QPL groups analyzed together for these measures)   - 95% read QPL - 75% used QPL during consultation   QPL Appraisal (self-reported in post-consultation questionnaire, both QPL groups analyzed together for these measures)   - 66% found it useful & thought it would be useful in future consultations   Consultation duration: significantly longer in both QPL groups compared to control group QPL 45, Combined 43, Control 36, p=.028  Anxiety: No group differences in Hamilton Anxiety and Depression Scale QPL 3.6 (3.2), Combined 4.5 (3.1), Control 3.9 (3.4), p=0.73  Depression: No group differences in Hamilton Anxiety and Depression Scale QPL 2.5 (2.9), Combined 2.6 (2.5), Control 2.0 (2.6), p=0.38  Patient satisfaction with information retrieved and doctor relationship: No group differences in Cancer Patient Experiences Questionnaire  Quality of Life: No group differences in European Organization for Research and Treatment of Cancer Quality of Life-C30 Questionnaire QPL 67 (19), combined 70 (20), control 64 (20), p=0.27 |
| Hjelmfors 2018 **(42)**  Sweden  Heart failure | To develop and pilot test the feasibility, acceptability and efficacy of a communication intervention among cardiology clinicians | Questionnaire | Sample size: 16 clinicians (13 nurses and 3 physicians)  Sex: 100% female  Age: Mean 39  Education: 60% specialist education (nurse - cardiology, public health; physician - general or internal medicine). Mean 9 years working with heart failure patients | Number and type: 1 multifaceted   - Pre-formed QPL + clinician communication skills training (An online communication course for cardiology professionals, with a component on learning how to use a QPL as a tool to communicate about heart failure trajectory and end of life care)   **Pre-formed QPL Description**  Recipients: Clinicians, patients and family members  Personnel or setting: Cardiology clinic  Developers: Developed in part 1 of this study via multiple “brainstorming” sessions with patients, clinicians and researchers  Purpose: To function as an aid at clinical appointments, and encourage question-asking about heart failure trajectory and end of life care. Meant to function as a communication tool to help patients and families be more involved in care  Content: Questions arranged into topics (diagnosis, support, end of life, family member, medical devices); blank space for additional questions  Format: Printed A4 booklet  Delivery: Clinicians were instructed to use the QPL in their clinical work with one patient  Intensity/Duration: 7-page, 45 questions, 5 categories  Copy of QPL: Not reported (NR) | QPL Appraisal:   - 69% reported QPL would be useful in future clinical conversations - 69% felt that QPL could be shortened   Note: results are limited as the emphasis of this study was developing the intervention and half of the results pertained to acceptability/efficacy of the clinician communication skills course. |
| Hyatt 2018 **(43)**  Australia  Heterogeneous cancer | To explore low-English speaking patient perspectives, experiences, and preferences regarding use of a QPL and consultation audio recording | Qualitative interviews | Sample Size: 18 cancer patients  Age: Mean 61  Sex: 40% female  Education: 5% no formal schooling; 15% primary; 20% secondary; 45% tertiary; 12.5% trade; 2.5% missing  Group Differences: All participants were from the QPL + audio recording group of RCT (none from control) | Number and type: 1 multifaceted   - Pre-formed QPL + consultation audio recording (the clinic provides patients with an audio recording the patient’s consultation with their healthcare provider that they can listen to again at home to facilitate memory, understanding and clarification of information)   **Pre-formed QPL Description**  Recipients: Patients  Personnel or setting: Two city-based outpatient oncology clinics  Developers: Previously developed by this group of researchers and pilot tested among low English-speakers  Purpose: To improve low English-speaking patients’ communication with clinicians and participation in healthcare  Content: Questions covering diagnosis, prognosis, cause, treatment, nutrition, diet, finance, sexuality, transport, and complementary therapies. Available in 4 languages  Format: Print  Delivery: Given to patients by researcher in the waiting room upon arrival for appointment with verbal encouragement to read it and ask their clinician questions  Intensity/Duration: 1 double sided A3 page folded into A4 sized booklet. 77 questions.  Copy of QPL: Not in manuscript | Interviews revealed 4 themes related to QPL appraisal:  Useful Resource:   - Prompts questions forgotten or not considered - Should be available to all patients upon arrival - Especially to those with low education level or new diagnosis   Support:   - Helps during shock and confusion following diagnosis, before any initial consultation - Recommended adding condition specific questions (e.g., for those who just had surgery)   Potential cultural barriers:   - Discomfort asking questions, viewed as “wasting” interpreters’ or doctors’ time - Belief that only doctors (not patients) should ask questions - Belief that it’s more appropriate to rely on family to prompt question-asking - Good translation and cultural acceptability of questions in QPL (facilitator)   Tailored approach   - Viewed as comprehensive and general enough to use in many situations - Some questions viewed as not relevant/too simplistic for those diagnosed some time ago - Would be more useful if tailored to the individual - Insufficient time to read QPL before appointment - Perhaps better to use time to develop own questions |
| Jacobs 2018 **(44)**  The Netherlands  Oesophageal cancer surgery | Explore feasibility of a web-based QPL for patients undergoing surgery for esophageal cancer (pilot test) | Questionnaire | Sample size: 21 post-esophageal surgery patients; 3 surgeons  Age: Median 62 (patients)  Sex: 5% female (patients)  Education: Middle (13/21) to high (8/21) education levels (patients)  Internet experience: 90% had >5 years (patients) | Number and type: 1 single-component   - **Pre-formed QPL**   Recipients: Patients in collaboration with surgeons  Personnel or setting: First follow up consultation after esophageal cancer surgery. City-based surgical unit at a hospital.  Developers: Researchers, based on literature, expert opinion, usability testing, questionnaires and interviews with 48 patients  Purpose: To support information provision of health-related quality of life topics after surgery  Content **Instructions for use** (send copy to surgeon before consultation, print copy for use during consultation), example questions/topics arranged into 9 domains: surgery/hospital stay, future, physical activities, social/emotional problems, eating, probe, other care, physical problems and medical care  Format Web-based system with options to: build a QPL using example questions, modify using custom questions, select Top 3 most important questions, send copy to researcher/surgeon, export and print. Used colours with stark contrast (e.g., yellow and blue), large font, no scrolling, separate pages for each domain with previous and next buttons, overview page separates items with varying shades of blue so they are separated without suggesting rank or importance. The order of appearance of domains is randomized for each patient.  Delivery: Link to QPL and personal login code emailed 1-7 days before follow up consultation. Patients need to login with personal code, state their initials, enter date of birth, surgeon who operated on them, date of initial follow up consultation, whether they want to print the QPL and whether they want QPL to be sent to the surgeon by email prior to the initial follow up consultation.  Intensity/Duration: 75 example questions or topics  Copy of QPL: Detailed breakdown of features in (44) online supporting information. Copy of intervention not provided. | QPL use: *Pre-consultation*   - 95% sent to researcher - 95% saved QPL - 81% printed QPL - 33% selected the top 3 questions - 67% sent to surgeon - 36% received and read by surgeons before consultation   QPL use: *During consultation*   - 81% brought QPL to consultation - Median 20 questions selected - 77% of selected questions were addressed - Equal number of questions initiated by patients and surgeons - Surgeons mainly initiated questions about surgery, hospital stay & future - Patients mainly initiated questions about physical, social & emotional problems   Consultation duration: Median 20 minutes  QPL Appraisal: *Patients*   - 86% believed other patients would want to use QPL to prepare for follow up - 62% preferred online > paper - 81% believed QPL was: easy to use, understandable, well arranged, and helpful in preparing for consultation - 100% would recommend QPL to other patients - 86% would use it again   QPL Appraisal: *Surgeons*  Believed:   - QPL was helpful in 65% of consultations - Increased duration of 45% of consultations - QPL did not provide a better overview of patient information needs   Recommended:   - Sending QPLs to surgeons earlier (i.e., one day before consultation) - Removing all example questions (felt that they encouraged patients to ask as many questions as possible and weren’t actual patient concerns) - Top 5 instead of top 3 patient generated questions |
| Khan 2018 **(45)**  Australia  Polycystic ovary syndrome | Explore feasibility of a QPL for women with PCOS (pilot test) | Questionnaire | Sample size: 20 women with PCOS  Age: Mean 28  Education: 45% undergraduate degree, 25% no post-secondary education | Number and type: 1 single-component   - **Pre-formed QPL**   Recipients: Patients  Personnel or setting: Standard individual consultation with endocrinologist at outpatient clinic in city  Developers: Researchers, using existing patient and clinician information sheets, PCOS guideline, online survey of 249 women with PCOS, and multiple rounds of qualitative interviews  Purpose: To assist women with information seeking and targeted question asking  Content: QPL description, target audience, **instructions for use** (women should consult online resources for answers to general questions and choose situational questions to discuss with clinicians), questions in categories by topics and general versus situational, online evidence-based PCOS resources of varying literacy levels  Format: Printed booklet  Delivery: Mailed one week before consultation  Intensity/Duration: 22 pages, 169 questions  Copy of Intervention: Link provided in publication [https://www.monash.edu/__data/assets/pdf_file/0009/1401768/PCOS-QPL.pdf](https://www.monash.edu/__data/assets/pdf_file/0009/1401768/PCOS-QPT.pdf) | QPL use: 100% read it; 30% accessed online resources; 60% asked 1-2 questions; 20% asked several questions; 10% said it helped them think of their own questions  QPL appraisal: Most women strongly agreed that the QPL:   - Was helpful (95%) - Made it easier to ask questions, put their concerns into words, or ask/discuss difficult/sensitive issues (75%) - Increased confidence about managing PCOS symptoms (65%) - Would use QPL again (90%) - Would recommend QPL to other women (95%)   Anxiety: 95% of women did not feel more anxious or worried after using the QPL  Format: 55% reported they would prefer to access QPL through a website or mobile phone app |
| Arthur 2017 **(46)**  US  Advanced Cancer (palliative care) | To determine whether a single page QPL helps patients communicate with clinicians | Questionnaire | Sample size: 100 advanced cancer patients  Age: Mean 59.7, SD 13.1  Sex: 50% female  Education: Less than high school 2%; high school 16%; associate degree 29%; Bachelor’s degree 35%; Advanced degree 17%; other 1% | Number and type: 1 single-component:   - **Pre-formed QPL**   Recipients: Palliative care patients and caregivers  Personnel or setting: Outpatient palliative care city-based clinic  Developers: Expert panel of clinicians in Department of Palliative Care and Rehabilitation Medicine at University of Texas MD Anderson Cancer Center using a three-round Delphi process  Purpose: To help palliative care patients communicate with their doctors  Content: **Introduction** describing that this is a list of common questions people with life-threatening illnesses sometimes ask their doctors. **Instructions** to indicate the questions you would like to ask today and the doctor will do their best to answer them. **Questions divided into** **sections as follows**: questions about the palliative care team and services; symptoms, treatment and lifestyle; end-of-life issues (with further instruction that these questions may or may not be relevant); and for caregivers (further instruction that if you have a caregiver these questions may or may not be useful to them).  Format: A single printed page not folded, questions presented as numbered list, titles centered, list left aligned, titles bolded and italicized, black text white background  Delivery: Eligible participants were given the QPL by research staff and patients were encouraged to identify the questions that they would like to discuss with the physician. At the end of the visit, QPLs were collected unless participants asked to keep a copy.  Intensity/duration: single-page, 25-items divided into 4 sections **(with headings)**, 5-8 questions per section.  Copy of QPL: current study, Appendix A | Patient QPL use:  The top 5 most frequently selected questions were about physical symptoms and treatment  Patient QPL appraisal (70-90% of patients agreed that):   - QPL information was clear to understand - QPL amount of information was just right - QPL helped them communicate with doctor - They would recommend QPL to other patients   Physician QPL appraisal:  68% of physicians agreed that QPL was helpful to patients in communicating with doctors  Patient anxiety:   - 75% disagreed that the material made them anxious - Anxiety significantly decreased from pre to post consultation (State Trait Anxiety Inventory, 39.3 vs. 33.8; p<0.0001)   Patient satisfaction: 92% of patients were satisfied with consultation  Consultation duration: 73% of physicians agreed that QPL did not prolong consultation visit  Predictors of QPL helpfulness:   - Presence of a caregiver significant predictor of patient rating QPL as helpful (univariate analysis p=0.007; multivariate analysis p=0.015) - History of smoking significant predictor of patient rating QPL as helpful (univariate analysis p=0.04) |
| Bottacini 2017 **(47)**  Italy  Breast cancer | Investigate compare the impact of a pre-formed QPL versus patient-generated QPL | Randomized controlled trial | Sample size: 308 women with early-stage breast cancer; 20 oncologists  Age: Mean 55.6 (patients); Mean 38.8 (clinicians)  Sex: 100 female (patients); 85% female (clinicians)  Education: 19% primary school, 28% secondary school, 40.5% high school, 42.5% university (patients); mean 11.3 years as medical oncologist (clinicians)  Group differences  Group 1: Pre-formed QPL  Group 2: Patient-generated QPL | Number and type: 2 single-component interventions   - Pre-formed QPL - Patient-generated QPL   **QPL Description**  Recipients: *Both:* Patients  Personnel or setting: *Both:* Outpatient oncology clinic  Developers: *Both:* Researchers in Australia, adapted by this group of researchers for Italian patients  Purpose: *Both:* To increase the number of questions that early-stage breast cancer patients ask during their first encounter with an oncologist  Content: *Pre-formed*: Evidence based questions that patients commonly want to ask their oncologist; **instructions for use**; *Patient-generated:* **instructions** “please indicate the issues which you want to discuss today with your oncologist and the questions you want to ask”, **blank space for questions**  Format: *Pre-formed:* printed brochure, A5 sized page folded into 6-page brochure *Patient generated:* blank lined form  Delivery: *Both* Participants were given QPL in a private room in clinic. Instructions were to circle salient questions, if any, that they would like to ask their oncologist  Intensity/Duration: *Pre-formed:* 50 questions, 11 categories, **(with section headings)** 2 pages; *Patient-generated:* 1 page  Copy of QPL: *Pre-formed:* Cancer Institute NSW Sidney AUS. Medical/Radiating Oncologist Question list <https://www.cancer.nsw.gov.au/getattachment/27b46c49-b480-4517-90bc-cc6c1317b2af/2009-06-15_question_list_oncologist.pdf> | Consultation Duration: average 49 mins. Significant correlation between consultation length and number of questions asked (p<.01) across all groups  Questions asked:   - Mean 22 questions selected in QPL group (vs. mean 2.4 in PGQPL group) - QPL group asked significantly less of their selected questions (8% vs. 45%, p<.01) - No significant difference in overall number of questions asked between groups - The presence of a companion reduced the number of questions asked   Content of Questions Asked: No significant group differences. Questions were mainly about illness management and administrative issues  Satisfaction with information: QPL group significantly less satisfied with information than QL group (p<.05). QPL group reported less need for information than QL group (p<.05).  Satisfaction with treatment decision: No significant group differences  Anxiety: No significant group differences (decreased post consultation in both groups) |
| Eggly 2017 **(48)**  US  Heterogeneous cancer (breast, colon, or lung) | To compare the impact of a QPL alone versus QPL + coaching on communication related outcomes during interactions between black patients and non-black oncologists | Randomized controlled trial | Sample size: 114 black oncology patients; 18 oncology physicians  Age: Mean 58.9 (patients); mean 46.8 (clinicians)  Sex: 91.2% female (patients); 44% female (clinicians)  Education: 22.8% less than high school, 12.3% high school, 33.3% some college, 18.4% college, 13.2% post-graduate degree (patients); 83.3% attending (clinicians)  Group differences:  Group 1: Usual care (control)  Group 2: QPL only  Group 3: QPL + communication coach session | Number and type: 1 single-component, 1 multifaceted   - Pre-formed QPL - Pre-formed QPL + communication coach session (in person session with one of three black female research staff using the “GPS: generate, prioritize, summarize” coaching method to help patients decide on questions to ask 2 weeks before consultation)   **Pre-formed QPL Description**  Recipients: Black oncology patients  Personnel or setting: City-based hospital outpatient oncology clinic  Developers: Researchers in collaboration with black patients, caregivers, community members and oncologists. Designed to be accessible to patients with low levels of education and health literacy  Purpose: To increase active participation and communication related outcomes among black patients and non-black oncologists  Content: Questions about diagnosis, treatment, chemotherapy, side effects, daily life during treatment, treatment plan and schedule, costs, and coping  Format: Printed booklet  Delivery: QPL-only group received QPL 2 weeks before consultation with a verbal explanation and encouragement to read it, show it to friends and family, and bring it to visit  Intensity/Duration: 43 questions  Copy intervention: See previous research by this group (not in manuscript) | QPL Appraisal: No significant group differences, *both strongly agreed that QPL*:   - Was helpful - Made it easier to ask questions - Some questions were useful - Helped put concerns into words - Will be useful too in future - Questions were easy to understand   *Both groups strongly disagreed*:   - Questions made them uncomfortable - The booklet contained too many questions   Consultation duration: No significant difference between either intervention versus control  Active participation: QPL-only group rated as participating more actively than control (p=.06). QPL-only group made more active participation statements (asking questions, making assertions, expressing concerns) versus control (p=.02) and versus QPL-coach (p=.02). No sig. difference between QPL-coach group versus control.  Patient-centredness: No significant difference between QPL-only versus control. QPL-coach group perceived oncologists as significantly less patient centred versus control.  Shared decision making: No significant group differences  Trust in oncologist: No significant group differences |
| Epstein 2017 **(49)**  US  Advanced cancer | To investigate the impact of a combined intervention including physician communication skills training and a QPL for patients on patient-centred communication, shared understanding, patient-physician relationship, quality of life, and healthcare utilization at the end of life, compared to usual care. | Randomized Controlled Trial | Sample size: 265 advanced nonhematologic cancer patients; 38 medical oncologists  Age: Mean 64.4 (patients); Mean 44.6 (physicians)  Sex: 55% female (patients); 29% female (physicians)  Education: high school or less 28%; some college or more 72% (patients)  Group differences:  Group 1: QPL + patient and caregiver coaching + Physician communication skills training  Group 2: usual care | Number and type: 1 multi-faceted (3 components)  **Pre-formed QPL** + **patient and caregiver coaching** (30-45 minute in person session before consultation and 3 follow up phone calls after consultation with trained research staff on how to use a QPL to help patients bring their most important concerns to their oncologist’s attention) + **physician communication skills training** (2-session in person training using a brief video; scenario based feedback from standardized patients portraying roles of patients with advanced cancer; and feedback from standardized patients on 2 audio-recorded consultations)  Recipients: Patients and caregivers  Personnel or setting: City based community and hospital cancer outpatient clinics  Developers: NR  Purpose: Overall purpose of multi-faceted intervention was to facilitate patient-centred communication by engaging patients in consultations, responding to emotions, informing patients about prognosis and treatment choices, and balanced framing of positive and negative aspects of decisions  Content: Questions commonly asked by patients with advanced cancer about: illness, available treatments, palliative care, psychosocial issues, impact on family and advance directives.  Format: print  Delivery: Given to patients by a coach 60 minutes before consultation, along with 30 to 45-minute coaching session.  Intensity/duration: NR  Copy of QPL: Not provided. Briefly described QPL in online Supplement 3, page 9. | Patient-centred communication (score derived from scale that considers patient-clinician relationship, exchanging information, managing uncertainty, making decisions, responding to emotions, and enabling self-management): significantly increased in QPL group vs. control (p=0.02) |
| Rodenbach 2017 **(50)**  US  Advanced cancer | Investigate the impact of QPL on number and nature of topics brought up in the oncology office visit | Randomized controlled trial | Sample size: 170 patients, 24 oncologists  Age: Mean 64.6 (patients); Mean 44.5 (clinicians)  Sex: 59.5% female (patients); 25% female (clinicians)  Education: 64.5% college+ (patients); 17% breast cancer specialist (clinician)  Group differences:  Group 1: QPL  Group 2: usual care | Number and type: 1 single-component:   - **Pre-formed QPL**   Recipients: Patients and caregivers  Personnel or setting: Outpatient oncology clinic, city-based,  Developers: Adapted from QPL developed by researchers in Australia, refined by this group via focus groups and interviews with advanced cancer patients  Purpose: To improve patient-centred communication based on 6 core PCC functions identified by the National Cancer Institute  Content: Questions in categories; and individual coaching with social worker to review QPL, identify/prioritize 2-3 topics of interest, and review how to ask questions/express concerns during consultation  Format: Printed booklet  Delivery: received QPL and coaching on same day as consultation  Intensity/Duration: 33 questions organized into 9 topics **(headings provided)**  Copy of Intervention: Current study Table 1 (49) | Number of topics discussed:   - 70.2% of QPL patients initiated QPL-related topics during consultation (vs. 32.6% in control, p<.001) - 99.3% of 140 initiated topics were discussed with oncologist in QPL group; 100% of 55 initiated topics were discussed with oncologist in control group   Content of topics discussed:   - QPL patients brought up more topics related to expectations/prognosis than control (p=.02) - QPL patients brought up topics that spanned the entire booklet (vs. 33% QPL-related topics never discussed in control group) |
| McLawhorn 2016 **(51)**  US  Advanced cancer (palliative care) | To evaluate the impact of a prognosis-focused QPL compared to usual care on patient-provider dialogue about Do Not Resuscitate (DNR) orders and hospice referrals. | Non-randomized Controlled Trial | Sample size: 294 cancer inpatients (46% lung)  Age: Mean 63.1  Sex: 52.7% female  Group differences:  Group 1: Pre-implementation of QPL into routine care  Group 2: Post-implementation of QPL into routine care | Number and type: 1 single-component   - **Pre-formed QPL**   Recipients: Patients with incurable cancer  Personnel or setting: Oncology inpatient units, tertiary care unit, city  Developers: This research group compiled a list of questions from previously published, relevant QPLs, obtained from the literature. Then they consulted with laypersons, physicians and advanced practice nurse peers to review the QPL for understandability and clarity. The QPLs used were developed via content analysis of focus groups and patient interviews performed by researchers  Purpose: To promote patient-provider dialogue about prognosis for incurable cancer  Content: Broad, open-ended questions about prognosis for incurable cancer  Format: Printed out and included in an admission packet given to all patients. The package included a welcome letter, a list of telephone contact information, and other informational brochures and cancer guides.  Delivery: Given to patients during admission to the inpatient oncology units. Patients kept the QPL along with all other documents in their admission packet.  Intensity/duration: 15 questions, 1 page  Copy of QPL: Included in article, page 386 | Do not resuscitate orders: Increase in active DNR orders (24% pre- vs. 39% post- QPL implementation)  Hospice referral: Increase in hospice referral rate (13% pre- vs. 22% post- QPL implementation) |
| Walczak 2015 **(52)**  Australia  Advanced Cancer (palliative care) | To explore patient and caregiver perspectives on QPLs included in a communication support program, and their openness to discussing prognosis, end of life issues, and advance care planning. | Qualitative Content analysis of patient responses in communication support program | Sample size: 31 patients with advanced cancer (19.4% breast), 11 corresponding caregivers  Age: Mean 63 (patients), Mean 62.2 (caregivers)  Sex: 45.2% female (patients); 80% female (caregivers)  Education: 16.1% Less than high school, 16.1% high school, 54.9% post-secondary; 12.9% post-graduate (patients)  12.9% Less than high school, 6.5% high school, 12.9% (caregivers) | Number and type: 1 multi-faceted   - Coaching (one 60 to 90 minute in person session one week before consultation, and one follow up phone call. Nurses delivered the coaching by introducing the patient and caregiver to QPL, exploring the QPL in depth, and discussed end of life care content and prognosis) + Pre-formed QPL   Recipients: Patients with advanced cancer (2 to 12-month life expectancy) attending an oncology consultation, and their caregivers  Personnel or setting: Six city-based oncology treatment centres  Developers: Developed and pilot tested in previous research by this group (55)  Purpose: To increase autonomous motivation to discuss prognosis and end of life care preferences; and increase competence to undertake decisions related to advanced cancer  Content: Questions about prognosis, treatment options/decisions, palliative care, lifestyle, patient/family support, advanced care planning, and caregiver-specific issues.  Format: A printed booklet with questions divided into sections  Delivery: A trained nurse discussed the QPL in detail with patient/caregiver one week before consultation, and encouraged them to choose questions for their next consultation.  Intensity/duration: NR  Copy of QPL: See (55) (4 pages, 9 Sections, 44 questions) | Analysis of transcripts by researchers using Likert scale ratings  QPL use   - 55% of patients expressed strong intent to use QPL immediately or in the future - 48% were highly engaged during session - 35% exhibited annoyance, uneasiness, or disinterest with QPL coaching - 13% had emotional responses to QPL (sadness)   QPL appraisal:  Facilitators to QPL use (Positive response to QPL associated with:)   - High information needs (multiple QPL questions selected) - Involvement in care (e.g., previous use of a QPL) - Being ready to discuss end of life issues (those who were direct in discussing imminent death in the consultation) - Realism and non-avoidant coping style re: end of life issues - Perception of appropriate timing for end-of-life discussion   Usefulness of content   - 45% of patients and caregivers did not want to discuss life expectancy and thought these questions had low utility (too uncertain to be meaningful, answers often inaccurate, makes doctors uncomfortable) - 61% of patients and caregivers were interested in discussing advanced care planning |
| Brandes 2014 **(53)**  The Netherlands  Advanced cancer (palliative care) | To investigate advanced cancer patients and caregivers’ appraisal of a QPL compared to usual care. | Randomized Controlled Trial | Note: details only reported for QPL group  Sample size: 28 advanced cancer patients (18% breast)  Age: Mean 63, SD 11  Sex: 54% female  Education: 14% less than high school; 14% high school; 50% post-secondary; 22% post graduate  Group 1: QPL + coaching  Group 2: usual care | Number and type: one multi-faceted   - Pre-formed QPL + coaching (1 in-person session between patient and trained nurse 2-3 weeks prior to the consultation to discuss which questions patients would like to ask, barriers and facilitators to asking questions; and to provide information regarding end-of-life care. And 1 follow up telephone session 2 weeks after the consultation to discuss QPL usage)   **Pre-formed QPL Description**  Recipients: Patients with advanced cancer who are still seeing their medical oncologist and their caregivers  Personnel or setting: Four city-based hospitals with cancer patients with 2-12 months of life expectancy  Developers: NR  Purpose: To encourage and facilitate greater discussion of prognosis and end-of-life care.  Content: **Title** (Asking questions: What now and what next…); **Introduction** (explains the importance of question asking in the context of cancer, and has **instructions for use** indicating to circle questions they want answered and to write additional questions in the blank space provided); **9 sections with 3-6 questions** per section, including: my cancer and what to expect in the future, my treatment options, palliative care, making a decision, my lifestyle, support for me, support for my family, making sure my wishes are honoured, other questions your family and/or caregiver may like to ask; **blank space** for additional questions  Format: Printed page(s), organized into sections (see content above)  Delivery: Provided to patients 2-3 weeks before consultation by trained nurse during coaching session where QPL was discussed (see above).  Intensity/duration: 39 questions, 9 sections **(with headings)**  Copy of QPL: Appendix A | Elicited in unstructured follow up phone calls from nurse:  QPL Use:   - 100% of 19 patients who were asked read QPL since coaching session - 66% of 21 who were asked used QPL in their last consultation   QPL Appraisal   - 85% of 13 patients who responded found QPL useful - 80% of the 10 who were asked planned to use QPL again in the future   Analysis of consultation audio recordings:  Number of questions asked (within QPL group):   - Range 2-30, Median 15 per consultation - Male participants asked more questions than female participants during consultation (median 10 vs. median 6, non-significant) - Female participants expressed more concerns during consultation (median 6 vs. median 4, non-significant)   Content of questions asked (within QPL group):   - 28.4% of questions asked and concerns expressed coincided with QPL questions - 99.1% of patients altered wording of QPL questions – tailored to their personal situation and/or reframed to be more optimistic/direct - Largest proportion of QPL questions asked were about disease and prognosis (56.6% for patients; 60% for caregivers) - The largest proportion of non-QPL questions asked were about symptoms (34.7% for patients; 27.3% for caregivers) and treatment (25.4% for patients, 54.5% for caregivers)   Questions planned for next consultation: Largest proportion were about treatment (25.4% for patients), prognosis and disease state (22% for patients), and test results (22% for patients) |
| Hamann 2014 **(54)**  Germany  Depression | To compare the effects of a QPL on active patient behaviour during consultations with usual care for outpatients with depression | Randomized Controlled Trial | Sample size: 100 outpatients with depression (72% recurrent depressive disorder)  Age: Mean 46.1  Sex: 30.5% female  Education: 6% <9 years, 31% 9 years, 31% 10 years, 29% 12< years  Group differences:  Group 1: QPL  Group 2: Usual care | Number and type: 1 single-component   - **Pre-formed QPL**   Recipients: Outpatients with depression  Personnel or setting: A large city-based psychiatric practice  Developers: Two psychologists and two psychiatrists described as “experienced clinicians.” QPL content was compiled from depression treatment guidelines and previously published QPLs. The draft was pilot tested and revised in the same psychiatric practice as this study  Purpose: To increase active patient behaviour in consultations  Content: **Instructions** to behave actively in the consultation by writing down notes about their wishes, and tick up to 15 questions on the QPL; 15 questions such as “what is my diagnosis”, “what treatment options are still available for my complaints” etc.); and a statement that patients could refer to the leaflet during the consultation.  Format: One-page printed sheet  Delivery: Provided to patients immediately before the consultation by a research assistant. Patients were asked to complete the QPL in a separate room with the research assistant present to help in case patients had questions.  Intensity/duration: 1 page, 15 questions  Copy of QPL: Appendix A (note QPL is in German) | QPL use (within QPL group):   - 100% read and worked through QPL - 71% made notes on QPL - 92% ticked 1+ questions   Number of Questions Asked (within QPL group):   - Questions ticked, Mean 3.7, SD 2.5 - Positively correlated with: German language ability (r=0.40, p=0.004), symptom severity (r=0.31, p=0.03), and participation preferences (r=0.38, p=0.02)   Content of Questions Asked (% of participants selecting QPL questions about each topic)   - 49% of patients selected questions about sleep disturbances - 47% change in medication - 45% strategies to increase quality of life - 43% coping with worsening of affective symptoms - <1% working or living conditions   QPL appraisal (within QPL group): 29% of patients rated QPL not helpful, 71% rated it somewhat helpful or helpful  Answers from post-consultation questionnaire:  Patient & physician satisfaction with consultation: No significant group differences (patient: QPL 5.0 vs. control 5.0, p=0.16; physician: QPL 3.0 vs. 3.0, p=0.46)  Patient & physician perception of shared decision making: No significant group differences (patient: QPL 3.0 vs. control 3.0, p=0.84; physician: QPL 3.0 vs. control 3.0, p=0.31)  Consultation duration: No significant group difference (QPL 8.56 min. vs. control 7.16 min., p=0.09)  Number of questions asked by patients: no significant group differences (QPL 3.5 vs. control 2.9, p=0.13)  Number of questions addressed by physicians: No significant group differences (QPL 5.9 vs. control 5.5, p=0.85) |
| Yeh 2014 **(55)**  US  Advanced Cancer | To examine the impact of a QPL examine how the QPL would be used and whether it would be well received by patients and health care providers. | Questionnaire | Sample size: 30 patients with advanced or metastatic head and neck cancer  Age: Mean 57.2, SD 13.5  Sex: 33% female  Education: 20% less than high school, 27% high school, 30% post-secondary, post-graduate 24% | Number and type: One single-component:   - **Pre-formed QPL**   Recipients: Patients with advanced cancer attending a new-patient consultation  Personnel or setting: City-based comprehensive cancer center outpatient clinic  Developers: Developed in the current study by researchers compiling and simplifying/shortening relevant questions and issues from two previously published interventions: a 20-page QPL on palliative care; and a short form on cancer treatment (without palliative care).  Purpose: To help patients get information about their treatment and quality of life  Content: **Introduction** explaining the purpose of the QPL (3 sentences); **Instructions for use** (take a few minutes to read the form, indicate questions you’d like to discuss and write down other questions in blank space, you can keep the sheet with you when seeing the doctor), **initial set of questions about cancer**, and **another section with end-of-life questions** with a statement that these may or may not be relevant; **blank space** for additional questions.  Format: A single printed page  Delivery: Provided to patients in the waiting room before consultation by a research staff, with verbal encouragement to use it however they wished. Physicians were not instructed to endorse QPL.  Intensity/duration: One page, 12 questions, 2 sections  Copy of QPL: Table 1 (page e138) | Information Needs:   - 77% of patients wanted as many details as possible about medical care - 83% of patients wanted both good and bad news about medical care   Decision making preferences:   - 57% desired active role in decision making with oncologist - 43% desired shared decision-making role with oncologist   Patient anxiety:   - State Anxiety Scale score decreased post- QPL use (Mean 46.0 pre- vs. Mean 39.6 post-consultation, p<0.005) - 80% disagreed that QPL questions made them feel anxious - 70% disagreed that QPL felt overwhelming to read   Patient satisfaction with consultation: Patient Satisfaction with the Consultation Scale scores were high, mean 113.1, median 111.5 (scores range from 25 – 125)  QPL Use (patients):   - 93% fully read and understood QPL - 90% used QPL during consultation - None chose to share QPL with oncologist   QPL Appraisal (patients):   - 97% found QPL easy to understand - 90% found QPL relevant - 90% found QPL helpful to themselves - 83% found QPL helpful to their caregivers - 90% would recommend QPL to patients and clinicians   QPL Appraisal (physician):  One physician reported no negative impact of QPL on workflow or consultation, and noted that patients did not share QPL with her |
| Walczak 2013 **(56)**  Australia  Advanced Cancer (palliative care) | To explore patient and physician’s culturally-specific (US and Australia) perspective on using a QPL for planning end-of-life care. | Qualitative interviews and focus group with selected interview participants | Sample size: 34 patients with advanced cancer (60% lung) and corresponding physicians and nurses (details NR)  Age: Mean 62.8  Sex: 44.2% female  Education: 17.9% Less than high school, 19.8% high school, 52.9% post-secondary; 6% post-graduate  Group differences:  Group 1: Australian QPL  Group 2: US QPL | Number and type: Two single-component:   - Pre-formed Australian QPL - Pre-formed US QPL   Recipients: *Both:* Patients with advanced cancer and oncology and palliative care doctors and nurses.  Personnel or setting: One US and two Australian treatment centers  Developers:  *Australia* A 7-member expert panel (health communication researchers, psychology, medical oncology, palliative care, critical care nursing and family medicine professionals), selected relevant literature and compiled relevant questions from existing QPLs obtained via database search. If an existing question didn’t exist for a topic of relevance, a new question was drafted. The expert panel then decided on order of questions, drafted other QPL components, and reviewed the prototype for readability and acceptability in the Australian setting.  *US* The Australia QPL draft was reviewed by researchers in the United States for cultural appropriateness in their setting. They reordered questions, removed questions and sections, and changed Australian expressions to US expressions.  Purpose: *Australia:* To help patients get the information that they want about: their illness and what they can expect in the future; *US:* To help doctor’s focus on patient concerns.  Content: *Australia:* **Title**, **Introduction and instructions** for use (6 paragraphs covering purpose of QPL, importance of question asking, detailed instructions about circling relevant questions, writing questions in blank space, using booklet as a checklist, using it during different consultations for different reasons etc.), **Questions in sections** about prognosis, non-curative treatment options, decisions, end of life issues, advanced care planning, supportive or palliative care and services for the patient, caregiver or family, **blank space** for other questions. *US:* Title, **Introduction and instructions** for use (1 paragraph, includes purpose, and instructions to circle 3-4 most important questions, ask them early in the visit, and add any questions of their own); **questions in sections** about the same topics as Australia QPL, **blank space** for additional questions  Format: Printed booklet  Delivery: n/a (similar to references 40,65)  Intensity/duration: *Australia*: 4 pages, 9 sections, 44 questions; *US:* 4 pages, 9 sections, 27 questions **(Section headings are present)**  Copy of QPL: Both in Appendix 1 (Online supplemental material) | QPL Appraisal:  Helpfulness   - Patients & clinicians found QPLs helpful with: patient information needs when doctor is not an optimal communicator, and patient memory of questions - Patients found QPLs helpful with: managing information overload, prompting consideration of new issues - Patients thought QPL was useless for some questions such as spiritual and epistemological questions because doctors don’t have the answers   Content   - Patients & clinicians found content appropriate and comprehensive - Patients and clinicians in the US felt end of life questions were good but not relevant to them due to various reasons (e.g., time, coping style, outlook) - Clinicians felt that there should be instructions saying that questions can be addressed over several visits not just one visit   Timing: Participants wished they had QPL when cancer incurability was first disclosed to them  Cultural differences in appraisal of QPL content:  US clinicians had stronger opinions about which questions were appropriate to ask an oncologist than Australian clinicians (e.g., questions about support services should be directed at nurse or social worker) |
| Aranda 2012 **(57)**  Australia  Heterogeneous Cancer (breast, gastrointestinal, and hematologic) | To assess the impact of the ChemoEd education program (a combined intervention including a QPL) at time of first treatment and over time, compared to usual care. | Randomized Controlled Trial | Sample size: 192 cancer patients (43.5% breast)  Age: Mean 52.5  Sex: 65% female  Education: 38.5% High school or less; 21% Vocational; 37% University; 3.5% Not stated  Group differences:  Group 1: ChemoEd intervention  Group 2: Usual care | Number and type: Five multi-faceted interventions grouped together titled “ChemoEd Intervention” Presented in order of delivery to patients:   - #1 Pre-chemotherapy Education: **3 Plain language summaries** (1. Educational DVD about chemotherapy; 2. Printed one-page drug information sheet with sensory and procedural information; 3. One-page self-care sheet on 16 topics related to lessening chemotherapy side-effects) + **Pre-formed QPL** - #2 Pre-chemotherapy Education part 2: **Coaching** (in person education session) + **Plain language summaries** (chemo information book, drug information sheets, self-care brochures) + **tour** of Chemotherapy Day Ward - #3 Post cycle one coaching: **Coaching** (phone call about self-care and discussion about chemo cycle 1) - #4 Pre-cycle 2 intervention: **Coaching** (review patient issues in person and coaching about self-care)   **Pre-formed QPL Description**  Recipients: Patients with breast, gastrointestinal and hematologic cancers, receiving first ever course of chemotherapy  Personnel or setting: A city-based specialist cancer hospital  Developers: NR  Purpose: To facilitate tailored education  Content: NR  Format: print  Delivery: Given to patients by a nurse between 8 – 14 days before commencing chemotherapy, as part of a larger intervention  Intensity/duration: NR  Copy of QPL: NR | Information and support needs:   - No significant between-groups difference of psychological concerns about treatment, after intervention #1 which includes QPL (Cancer Treatment Scale, p=0.56) - Significant increase in intervention group procedural concerns about treatment vs. control post-intervention #1 (Cancer Treatment Scale p=0.02) - Significant decrease in psychological (p=0.027) and procedural (0.03) treatment concerns post intervention #4 (Cancer Treatment Scale)   Anxiety and depression: Hospital Anxiety and Depression Scale score non-significantly increased from baseline to intervention 1 which includes QPL (p=0.33) and from intervention #1 to intervention #2 (p=0.15)  Symptoms: Significant decrease in bother caused by, presence of, and severity of vomiting in intervention vs. control post intervention #4 (p=0.001) |
| Dimoska 2012 **(58)**  Australia  Heterogeneous cancer | To monitor the uptake of QPLs by patients in four cancer services; measure views of patients and health professionals on utility of QPL; and identify barriers and facilitators to implementation of a QPL. | Questionnaire and qualitative researcher observations | Sample size: 139 patients with cancer (48.2% breast); 20 physicians (10 medical oncology, 7 radiation oncology, 3 palliative care)  Age: Mean 59.7, SD 11.6 (patients); 70% aged 25 – 45 years old, 25% 46-55; 5% 56+ (physicians)  Sex: 65.5% female (patients); 55% female (physicians)  Education: 43.2% Less than high school, 17.3% high school, 14.4% tertiary non-university, 16.5% undergraduate, 5.0% post-graduate; 50% 1-5 years in specialty, 30% 6-15 years, 15% 16-25 years, 5% 25+ years (physicians)  30% of clinicians had experience with QPLs | Number and type: 3 single-component:   - Pre-formed QPL - radiation oncology + plain language summary (general information about the cancer centre) - Pre-formed QPL – surgeon - Pre-formed QPL – palliative care   **Pre-formed QPL Descriptions**  Recipients: Patients with heterogeneous cancer of any diagnosis and stage, seeing either a radiation oncologist, surgeon, or palliative care clinician.  Personnel or setting: The large cancer centres with high patient volume from two rural and two urban areas  Developers: *All*: Previous research by this group using focus groups and interviews with patients and health professionals (development) and randomized controlled trials (evaluation) (not in manuscript).  Purpose: To encourage patient participation during consultations and assist patients in acquiring information suited to their needs at their own pace  Content: *Radiation oncology and surgery:* Page 1 **Title** “so you have cancer…questions to ask your…(oncologist or surgeon)”; Page 2 – 4 **Introduction** (2 paragraphs); **Instructions for use** (1 paragraph); **Questions** about: how and when to ask questions, diagnosis, tests, prognosis, optimal care, multi-disciplinary team, treatment information and options; Page 5: **Blank space** for additional questions (one line instruction provided here too); Page 6: Instruction to keep the QPL for future use and **affiliations**; *Palliative care:* Page 1: **Title**; Page 2: **Affiliations and copyright**; Page 3: **Table of contents**; Page 4-5 **Introduction and instructions for use;** Majority of booklet: **Questions** divided into many sections and subsections. At the end of each section is a **blank space** for additional questions; Last page: **blank space** for “other questions” with one-line instruction.  Format: *Radiation and Surgery:* A5-sized, double sided brochure in hard copy, very light blue background throughout, majority of text in black, titles and section headings alternating between purple and red font, first and last page use white font on purple or red background *Palliative care:* A5 sized pages in a booklet published by University of Sydney Medical Psychology Research Unit, pages without questions have green background with white font, pages with questions or blank space have white background with black font and green borders.  Delivery: *All:* volunteer hospital staff handed out QPLs to patients in waiting rooms.  *Radiation oncology and surgery:* Reception staff either: mailed out QPL to new patients prior to first appointment, gave QPL to patients in person pre-consultation during registration, or gave QPL to patients in person post-consultation to be used in follow-up consultation.  *Palliative care:* QPL given to patients by palliative care clinicians during consultation or at admission to the inpatient unit.  Intensity/duration *Radiation oncology:* 6 brochure pages, 7 sections, 6 subsections, 49 questions *Surgery:* 6 brochure-sized pages, 7 sections, 5 subsections, 48 questions *Palliative care:* 16 A5 sized pages, 9 sections **(Section headings provided)**, 12 subsections **(subsection headings provided)**, 112 questions  Copy of QPL: *All* the University of Sydney Centre for Medical Psychology and Evidence-Based Decision-Making Website, Our Resources, QPL section: https://sydney.edu.au/science/our-research/research-centres/centre-for-medical-psychology.html | **Patients**  QPL Use   - 91% of patients approached accepted the QPL - 88.5% reported reading QPL - 44% reported referring to QPL in consultation at least once - 9% reported referring to QPL frequently   QPL Appraisal (helpfulness)   - 41% reported QPL helped them ask clinician more questions than they would have ordinarily asked - 44% reported QPL made it easier to discuss “difficult” or “sensitive” issues with oncologist - Of patients who read QPL, 92% believed it was useful for someone seeing a cancer specialist; and 80% would definitely or probably use QPL again - 84.5% of patients did not find QPL distressing   QPL Appraisal (Patient barriers to QPL use): 9% of patients declined QPL because lack of interest, all questions were already answered, felt overwhelmed, had too much on their mind, just came in for a check-up, too far along in illness to need to ask questions, belief that clinician would tell them everything they needed to know without having to ask questions  **Clinicians**  QPL Use: 48% of clinicians approached agreed to implement QPL into their practice  QPL Appraisal (Clinician barriers to QPL use): 52% clinicians declined implementing QPL because of potential negative impact on patients, consultation structure, consultation duration; and “good clinician” should already answer these questions without prompt  QPL Appraisal (pre-implementation expected helpfulness):   - 50% thought patients would use QPL - 30% thought QPL would make communication “very much” easier - 70% thought it would make communication “somewhat” easier - 85% thought QPL would impact how they provide information to patients - 85% thought QPL would help patients initiate discussions with clinicians   QPL Appraisal (post-implementation actual helpfulness):   - 60% reported QPL made communication easier - 60% reported QPL helped patients initiate discussions about sensitive topics - 100% believed QPLs are feasible to implement as part of routine cancer care and that this would not strain resources - 80% reported difficulty remembering to give QPL to patient or asking about it during consultation (barrier) - 100% would report patients to use QPL in future - 90% would recommend QPL to other clinicians - 60% believe QPLs are more suitable to new patients   Clinician identified facilitators to QPL dissemination: reliable supply of QPLs, reminding administrative staff to give QPL to patients, giving patients more time to read QPL before consultation, distributing QPL via mail prior to consultation, QPL translated to other languages  Clinician perceived patient barriers to QPL use: Clinicians thought patients didn’t use QPLs because they: were mostly satisfied with consultation and didn’t have further questions, not enough time to read QPL before consultation, too scared, uncomfortable, or embarrassed to ask questions, not interested, non- English speaking  Consultation duration: 70% of clinicians reported that QPL did not affect consultation length, 30% reported that it made consultation somewhat longer  **Staff**  QPL Appraisal (staff barriers to QPL use)  22% of patients at participating clinics didn’t receive QPL, reasons for staff not offering it to them included: staff forgot, were too busy, patient may be distressed by QPL, lack of time and staff, logistic issues  **Researcher**  QPL appraisal (implementation): researcher observed several factors to successful implementation including: education about QPL; active clinical champions with high level of authority and respect from peers; and involvement of staff at several points in process (choosing dissemination procedures, flexibility during execution of processes) |
| Langbecker 2012 **(59)**  Australia  Cancer (Brain) | To develop a brain-tumour specific QPL and pilot test its acceptability along with feasibility of outcome assessment within this population compared to an informational brochure. | Non randomized controlled trial | Sample size: 20 brain cancer patients (40% glioblastoma)  Age: Mean 51 years  Sex: 40% female  Education: 55% high school or less, 15% trade or certificate, 30% university  Group differences:  Group 1 (n=7): QPL + Plain language summary  Group 2 (n=8): Plain language summary alone (control) | Number and type: 1 multi-faceted and 1 single-component:   - Pre-formed QPL + plain language summary - Plain language summary alone (informational brochure about brain tumours)   **Pre-formed QPL Description**  Recipients: Patients with a brain tumour  Personnel or setting: Four city-based hospitals  Developers: Current researchers and study, using healthcare intervention development principles from O’Donnell and Entwistle 2003 (not in manuscript). Process involved: drafting QPL from thematic analysis of existing resources, refining QPL using feedback from patients and caregivers, readability assessment and redrafting, QPL reviewed by health professionals, QPL format designed and tested with patients and caregivers.  Purpose: To help patients get the information and support they need.  Content: Readability score of grade level 4.8 (Flesch-Kincaid). **Title page** “It’s okay to ask”; **Introduction** (2 paragraphs); **instructions for use**, **page numbers, table of contents,** **questions** (initial general set, then multiple sections with more detailed questions that may or may not apply to the patient – this is explained in instructions), **blank space** after each section for “other questions or note”, last page with **affiliations** (logo on first page too)  Format: A5 sized booklet, provided in hard copy, first page green background with black text and pictures of diverse patients (different ethnic and demographic backgrounds). The rest of the booklet is colour coded by section. First set is all white background with black text, next set is also white background black text but with green borders and title bolded in green colour, next set is the same with blue etc. Last page same format as first page. Pages clearly numbered, large font (looks about 14), white space around text.  Delivery: In person 4 weeks before consultation  Intensity/duration: 33 pages, 7 sections **(with headings)**, 21 subsections **(with headings)**, 189 questions  Copy of QPL: Appendix S1 (online) | Data from questionnaires, comparison to control provided where available, significance NR because participants were not randomized  QPL Use:   - 29% did not read QPL - 57.1% had enough time to read QPL (vs. 87.5% in control) - 40% preferred to have QPL at a different time - In qualitative discussion, patients mentioned wanting QPL at time of diagnosis   QPL Appraisal   - 100% agreed QPL was helpful (vs. 87.5% in control) - 86% agreed QPL made it easier to ask questions (vs. 75% in control) - 86% agreed that QPL helped them “put some of their questions or concerns into words” (vs. 87.5% in control) - 100% found QPL questions “useful” (vs. 87.5% in control) - 100% found QPL easy to understand (vs. 87.5%) - 70% thought the QPL will be useful to them in the future (vs. 87.5%) - 14% found QPL overwhelming (vs. 87.5% in control) |
| Shirai 2012 **(60)**  Japan  Advanced Cancer | To investigate patient perception of a pre-formed QPL compared to a patient-generated QPL. | Randomized controlled trial | Sample size: 63 advanced cancer patients (61.9% lung)  Age: Median 63.75  Sex: 33% female  Education: NR  Group differences:  Group 1: Pre-formed QPL and Patient-generated QPL  Group 2: Patient generated QPL alone | Number and type: 2 single-component:   - Patient-generated QPL - Pre-formed QPL   **QPL Descriptions**  Recipients: *Both:* Patients with advanced cancer attending first consultation with oncologist  Personnel or setting: *Both:* City-based, national cancer hospital  Developers: *Patient-generated QPL:* patients, details NR  *Pre-formed QPL:* Current researchers in current study. They drafted a QPL based on 1. Previously published QPLs in the literature; and 2. Their previous work on Japanese cancer patient preferences regarding disclosure of bad news. Then, they conducted qualitative interviews with patients and oncologists to review the QPL and make changes  Purpose: *Both*: To help patients decide on initial treatment (although questions in QPL were not solely about treatment options / decision making)  Content: *Patient-generated QPL:* **Introduction** with information on services at the hospital, facilitates, and treatment information. **Blank space** for questions or messages that patients may have wanted to write  *Pre-formed QPL* **Questions** about: diagnosis, condition of a disease, symptom, test, treatment, life, family, psychological issues, prognosis, other issues; **blank space** for new questions  Format: *Patient-generated:* Single printed page  *Pre-formed:* Hard copy booklet with A4 sized pages  Delivery: *Both:* Given to patients in person in an envelope by a researcher after initial consultation. Patients verbally instructed to read the materials before their next consultation  Intensity/duration: *Patient-generated:* One page; *Pre-formed:* 10 pages, 10 sections **(with headings),** 53 questions  Copy of QPL: *Patient-generated:* Not provided *Pre-formed*: Appendix A, Table A1 | QPL Use   - 75% read QPL (both groups) - 44% of patients in pre-formed QPL group decided on questions in advance (vs. 23% in patient-generated QPL, p=0.075) - 63% of patients in pre-formed QPL group asked questions, vs. 71% in patient-generated QPL (no significant difference)   Data from questionnaires where answers range from 0 – 10 rating, 10 = strongly agree  QPL Appraisal (usefulness)   - Usefulness of material in helping patients ask questions significantly higher in pre-formed QPL vs. patient-generated QPL (4.4+/-3.6 vs. 2.7+/-2.8, p=0.033) - Usefulness of materials in helping patients understand treatment plan higher in pre-formed QPL vs. patient-generated QPL (4.9+/- 3.6 vs. 3.3+/-2.8; p=0.051) - Willingness to use the material in future is significantly higher in pre-formed QPL vs. patient-generated QPL (5.3+/-3.8 vs. 2.8+/-2.8; p=0.006)   Patient satisfaction with consultation: No significant between group differences both high (Mean 7.9 in Pre-formed QPL vs. Mean 7.8 in patient-generated QPL, p=0.847)  Number of questions asked: No significant group differences, both groups Mean 1.0 question)  Content of questions asked: No significant group differences, both groups majority of questions related to information about treatment |
| Smets 2012 **(61)**  The Netherlands  Esophageal Cancer | To develop a QPL and pilot test its impact on patient question asking compared to usual care. | Randomized Controlled Trial | Sample size: 28 patients with esophageal cancer  Age: Mean 64.7  Sex: 25% female  Education: 48% low-level professional, 26% intermediate-level professional, 26% high-level professional  Group differences:  Group 1: QPL  Group 2: Usual care | Number and type: one single-component:   - **Pre-formed QPL**   Recipients: Patients with esophageal cancer.  Personnel or setting: Department of surgery at a city based Academic Medical Centre  Developers: Previous researchers developed and validated the QPL for surgical patients with various types of cancer (not in manuscript). This group translated it into Dutch using two translators, who compared, discussed and resolved any discrepancies. They also added questions about esophageal cancer patients' quality of life.  Purpose: To support patient memory, and help patients get information they want from their surgeons.  Content**: Introduction** about usefulness of question asking for patients and that question asking is welcomed by the surgeon. **Questions:** about diagnosis, tests, prognosis, treatment options, multidisciplinary team, surgery, effects of surgery, quality of life, and support information  Format: Single printed page, not much white space surrounding text. Not many details provided.  Delivery: Given to patients in person by a researcher 15 minutes before consultation  Intensity/duration: 38 questions, 6 sections **(with headings)**, 4 subsections **(with headings)**, 1 page  Copy of QPL: Appendix I | QPL Use (within QPL group)   - 88% returned QPL after consultation - Median 19 (of 38) questions were marked on QPL - Most patients marked one or more questions about surgery, the effects of surgery, quality of life, prognosis and treatment options - Few patients marked questions about the multidisciplinary team or questions about available information and psychosocial support - 78.5% indicated that partner/family also used QPL - 31% didn’t have enough time to complete QPL   Number of questions asked:  Significantly more questions asked in QPL vs. control (QPL Median 12, control Median 8, p<0.01)  Content of questions asked:   - Significantly more questions about treatment options, team and procedures in QPL group (Median 7 QPL, Median 4 control, p<0.01) - No questions (across groups) were asked about additional information sources or psychosocial care   QPL Appraisal:   - No significant group difference in “easiness to ask questions” (QPL Median 9 vs. control Median 8, p value NR) - Within QPL group, patients Median rating of contribution of QPL to information received as 7/10 - Within QPL group, patients would recommend QPL to others median 8.5/10   Consultation duration: No significant group difference (Median QPL 19 mins. vs. Control 14 mins., p value NR)  Patient satisfaction with consultation: No significant group differences (Median QPL 8.2 vs. control 8.2)  Patient satisfaction with independent time provided: No significant group differences (Median QPL 9 vs. control 8) |
| Lim 2011 **(62)**  Singapore  Surgery (Abdomen or Breast) | To assess the impact of a QPL on knowledge of pre and postoperative surgical care, and pre- and post-operative anxiety, compared to usual care. | Randomized Controlled Trial | Sample size: 230 patients scheduled for abdomen or breast surgery  Age: Mean 49, SD 9.6  Sex: 75.2% female  Education: 4.4% No formal education, 19.6% less than high school, 43.9% high school, 32.2% postsecondary  Group differences:  Pre- and post- operative anxiety measured in both groups  Group 1: QPL  Group 2: usual care | Number and type: One single-component   - **Pre-formed QPL**   Recipients: Patients scheduled for surgery  Personnel or setting: Surgical patients at a tertiary general hospital in Southeast Asia  Developers: Current study, researchers compiled the QPL from previously published resources – further detail NR  Purpose: A list of common questions which patients can use to seek clarification from surgeons regarding doubts about the operation and postoperative care, to hopefully reduce anxiety  Content: **Questions** about diagnosis, procedure, dangers/risks during surgery, recovery time, pain, how condition impacts work or other activities. Further details NR.  Format: Given to patients in printed form. Further details NR.  Delivery: Given to patients by research staff 1 – 3 weeks before surgery. Patients are able to ask surgeon questions in person, one day before surgery (during admission).  Intensity/duration: NR  Copy of QPL: Not provided. Excerpt from QPL provided on page 176, Table 1. | Anxiety (measured via State Trait Anxiety Inventory): QPL group had significantly bigger reduction in anxiety than control group at first post-operative follow up, (change of 20.4 QPL vs. 12.7 control; p=0.01)  Predictors of anxiety reduction (across QPL and control groups):   - Satisfaction with consultation (p=0.02) - Ability of doctor to answer all patients’ questions (p=0.035) - Leaving no unanswered questions about operation (p=0.029) |
| van Weert 2011 **(63)**  The Netherlands  Heterogeneous Cancer | To evaluate the effects of an educational intervention including a QPL on oncology nurse communication compared to usual care. | Randomized Controlled Trial | Sample size: 210 cancer patients (40% digestive-gastrointestinal); and 48 oncology nurses  Age: Mean 72.1 (patients); Mean 41 (nurses)  Sex: 34.8% female (patients); 95.3% female (nurses)  Education: 47.1% low, 17.6% middle, 15.5% high (patients); Mean 17.4 years of experience as a nurse  Group differences:  Both groups were tested before and after intervention  Group 1: Communication skills training + QPL  Group 2: Usual care | Number and type: One multi-faceted:  Clinician communication skills training (nurses underwent three sessions: one web-based video feedback where they watched a video of their own consultation and did a reflection exercise; and two in person sessions with a professional trainer to enhance knowledge and skills regarding patient education/communication about chemotherapy for older adults) + Pre-formed QPL  **Pre-formed QPL Description**  Recipients: Older adult patients with cancer  Personnel or setting: 12 wards of 10 hospitals  Developers: Collaboration between 2 research groups  Purpose: To encourage patients to acquire information that is personally relevant for them during a health care encounter and to enhance tailored communication  Content: **Questions** about 17 topics including, treatment, side effects, emotional topics, coping with illness, and sexuality.  Format: Printed booklet. Further details NR.  Delivery: Given to patients in person before consultation. Further details NR.  Intensity/duration: NR  Copy of QPL: Refers readers to website for more intervention details [www.nivel.nl/voice](http://www.nivel.nl/voice) | Consultation duration: No significant group differences (intervention: 56.83 mins vs. control: 57.37 mins)  Quality of communication (measured using the QUOTEchemo patient-centred chemotherapy communication questionnaire): Intervention group significantly higher in-patient ratings of tailored, affective, and interpersonal communication (p<0.01)  Number of questions asked (researcher observations of consultation): Patients and companions in QPL group asked significantly more questions (QPL Mean 10.76 questions vs. control Mean 6.69 questions; p<0.05)  Content of questions asked (researcher observations of consultation): QPL group asked significantly more questions about treatment than control (p<0.05). Overall, most questions were about hospital routines, details of therapy and side effects |
| Hebert 2009 **(64)**  US  Advanced Cancer (palliative care) | To develop a QPL for use by physicians and family members of patients in palliative care, and to pilot test the acceptability and feasibility of the QPL. | Questionnaire | Sample size: 56 caregivers of patients with advanced cancer  Age: Mean 53.7, SD 14.3  Sex: 73% female  Education: NR  Relationship to patient: 55% spouse, 45% non-spouse  Hours per week spent providing care to patient: Mean 132, SD 67 | Number and type: One single-faceted:   - **Pre-formed QPL**   Recipients: Caregivers of patients with advanced cancer, and palliative care physicians  Personnel or setting: Outpatient palliative care clinic in a National Cancer Institute designated centre.  Developers: Researchers in the current study. Ethnographic interviews were conducted with caregivers in their home or at the hospital, and focus groups were conducted with health care providers. The questions were compiled by researchers and the QPL was reviewed by the study team before pilot testing.  Purpose: To help caregivers discuss end of life issues with physician or nurse.  Content: **Title** (common questions asked by friends and family members); **introduction** (one paragraph); **instructions for use** (one paragraph explaining that some questions may or not apply, check off the ones you’d like to ask, doctors and nurses will do their best to answer); **questions** in two sections, general cancer questions and palliative care (labelled “when your loved one isn’t getting better…); **blank space** two blank lines for additional questions; **instructions** at the end saying to return the form to the doctor or nurse.  Format: Print with titles and **section headings** are in all capitals, questions and body text are normal case 12-point font, questions are presented as a check list, with questions on the left and a line to check off on the right,  Delivery: Given to caregivers by research staff in the waiting room before consultation, with verbal instructions to check questions they want to discuss with the doctor. Caregivers then gave the QPL to the physician.  Intensity/duration: 2 pages, 2 sections **(with headings)**, 25 questions  Copy of QPL: Appendix 1 | Answers to 5-point Likert Scale questions:  QPL Use (caregivers):   - 98% had enough time to complete QPL - 85% had enough time to discuss all their questions   QPL Appraisal (caregivers):   - 75% reported that QPL made it easier for them to ask questions - 78% felt comfortable discussing their questions in front of a family member - 100% found QPL easy to understand - 100% felt comfortable completing QPL in clinic - 85% liked the QPL - 11% thought QPL should be modified - 4% thought QPL added too much time to the visit   QPL Appraisal (physician):  Both of the 2 physicians participating verbally reported that QPL was a useful tool that did not detract from their clinical work |
| Clayton 2007 **(65)**  Australia  Advanced Cancer (palliative care) | To develop a QPL for advanced cancer patients and their caregivers who were referred for palliative care, and evaluate the effect of a QPL compared to usual care. | Randomized Controlled Trial | Sample size: 174 advanced cancer patients (38% GI)  Age: Mean 65.05  Sex: 39.6% female  Education: 28% less than 10 years, 39.5% 10 years, 13% 12 years, 9% tertiary non university, 10.5% tertiary university  Group differences:  Group 1: QPL  Group 2: usual care | Number and type: One single-component   - **Pre-formed QPL**   Recipients: Patients with advanced cancer and their caregivers  Personnel or setting: 9 specialist palliative care services in various settings (tertiary referral hospitals, district hospitals, inpatient units, homes, hostels, and nursing homes); majority outpatient palliative care clinics  Developers: This group in previous research (not in manuscript)  Purpose: To help patients get the information they want about palliative care and their illness  Content: **Title** (“Asking questions can help: an aid for people seeing the palliative care team”); Page 2: **Affiliations and copyright**; Page 3: **Table of contents**; Page 4-5 **Introduction and instructions for use;** Majority of booklet: **Questions** divided into many sections and subsections. At the end of each section is a **blank space** for additional questions; Last page: **blank space** for “other questions” with one-line instruction.  Format: Available in print for the study, afterwards only available online. A5 sized pages in a booklet published by University of Sydney Medical Psychology Research Unit, pages without questions have green background with white font, pages with questions or blank space have white background with black font and green borders.  Delivery: Patients randomly assigned to receive QPL from researcher either 20 or 30 minutes prior to consultation.  Intensity/duration: 16 A5 sized pages, 9 sections **(with headings)**, 12 subsections, 112 questions  Copy of QPL: University of Sydney Centre for Medical Psychology and Evidence-Based Decision-Making Website, Our Resources, QPL section, “Asking questions can help: an aid for people seeing the palliative care team”: https://sydney.edu.au/science/our-research/research-centres/centre-for-medical-psychology.html | Number of questions asked:   - Patients in QPL group asked 2.31 times more questions than controls (p<0.0001) - Caregivers in QPL group asked 2.11 times more questions than controls (p=0.0005) - Patients in QPL group raised significantly more issues than control (QPL Mean 12.7 items, control Mean 17.6 items, p=0.0002) - No significant difference in number of issues raised by caregivers vs. control   Content of questions asked:  QPL group asked more questions than control about:   - Palliative care service and team than control (QPL 4.5 vs. control 3.5 questions; p<0.0001) - Illness and what to expect in the future (QPL 3.1 vs. control 1.4 questions; p<0.003) - Lifestyle and quality of life (QPL 2.0 vs. control 1.29 questions; p<0.03) - Concerns about professional care (QPL 16 vs. control 1 questions; p<0.0001) - Caregiver issues (QPL 23 vs. control 5 questions; p<0.001) - End of life issues (QPL 27 vs. control 8 questions; p<0.0001)   Information needs: No significant group differences in unmet information needs, perception of too much information, or achievement of information needs  Patient anxiety (State Trait Anxiety Inventory): No significant group differences (24 hours after consultation: Mean 40.3 for both groups; 3 weeks after: Mean QPL 38.7 vs. Mean control 37.5)  Patient Satisfaction with consultation (score out of 125): No significant difference between groups (QPL Mean 110.1 vs. control Mean 110.3)  Clinician satisfaction with communication during consultation: No significant differences between groups  Physician QPL Appraisal:   - In 95% of consultations (out of 92 consultations) QPL did not interfere with consultation flow - In 46%, physicians believed that QPL was a useful tool, and would use it for future patients - 38% of physicians feared the patient may not be ready to discuss end of life issues, and were concerned that it would put onus on the patient to ask questions rather than physician to respond to patient cues   Physician endorsement of QPL: significantly, positively correlated with number of questions asked (p<0.0001). Number of patient questions in QPL group without physician endorsement similar to controls (QPL without physician endorsement mean 2.6 vs. control mean 2.3) |
| Hartmann 2007 **(66)**  US  Asthma | To develop a web-based QPL and understand its impact on the physician-patient relationship, patient-physician communication, and on asthma care. | Qualitative interviews | Sample size: 37 patients with asthma; 26 primary care physicians (demographic information NR)  Age: 21.6% between 21 – 35, 10.8% over 65.  Sex: 91.9% female  Education: 45.9% completed college | Number and type: One multi-faceted   - **“**Myexpertdoctor” website intervention**,** including: **Quiz** (patients had to answer 10-20 questions about their disease knowledge related to asthma and its care) + **Plain language summary** (the website generates personalized feedback and information based on patient answers to the quiz) + **Pre-formed QPL** (Questions are suggested that pair with the personalized feedback)   **Pre-formed QPL Description**  Recipients: Patients with asthma  Personnel or setting: Subjects were identified through a large health insurance company.  Developers: In previous research by this group (67) by reviewing evidence-based guidelines for suggested questions and then programming these questions into a website. They also selected and reviewed sites to include in the additional resources section. Funded by grants from the National Institutes of Health.  Purpose: To help patients ask the right questions, personalized to their particular condition and situation.  Content: **Title** “questions you should ask your doctor”; **Questions** personally tailored to patient needs which were assessed in initial quiz**; lay explanation** of why the patient should ask the physician each question, with justification related to their quiz answers; **Additional resources** a “more information” button that links to other websites for further reading and explanations of topics  Format: Web-based QPL. The website colours were shades of blue with black text and warnings in red (that arise from alarming answers to questions in the quiz section). Title and **section headings** in blue and larger font. Questions presented as a bullet form list at the top of webpage under title in 12-point font and blue colour because each question is a hyperlink that links to its corresponding section. Below the large question list is an individual section for each question, title in large blue font, with a lay explanation in black text for why the patient should ask that question, and a blue hyperlinked “more information” button in 12-point font.  Delivery: Patients could access the website up to 7 days prior to their next consultation, and email or telephone reminders to access the website were sent 7, 4, and 3 days before consultation by research staff. Patients were encouraged to print personalized feedback and QPL to bring to consultation.  Intensity/duration: Varies based on patient answers to quizzes. Based on examples provided could range from 0 – 6 questions, however maximum number of questions NR. All presented on one web-page where patient scrolls down to access more information (number of pages N/A). Each question and lay explanation presented as a new “section”  Copy of QPL: Example QPL in online Appendix | QPL Use (patients):   - 100% accessed website before visit - 72% brought a print out of website to consultation - 58% told the physician they had visited the website   QPL Appraisal (patients):   - 25% rated the website excellent, 55.6% rated it “very good” - 35%, especially those with “under control” asthma wanted more detail in both questions to ask and feedback - 27% wanted more *new* information (these patients were knowledgeable about the disease because they’ve had it for a long time / did their research) - 14% thought feedback wasn’t specific enough to their own situation   56% of patients said the website had influenced outcomes of their visit in some way, including:   - increased self-confidence in communicating with physicians - increased amount of time patients spoke during consultation - increased sense of security in what to ask physicians - increased confidence in their understanding of issues related to asthma and treatment - perceived improvement in patient-physician relationship - greater confidence in the care received - increased active involvement in care / knowledge of how to be involved in care - increased knowledge about the condition   QPL Appraisal (physicians):   - 11.5% rated the website excellent, 50% very good, and 23.1% good - 76.9% agreed that they thought the website would be useful in helping patients receive better health care - 14% suggested adding more visual aids - 23-45% suggested adding more specific information about medications and asthma |
| Ogawa 2007 **(67)**  Japan  Atopic dermatitis | To develop an atopic dermatitis QPL from the patients’ point of view, and investigate whether this would assist patients in bringing up questions they wanted to ask, and improve understanding of atopic dermatitis. | Questionnaire | Sample size: 30 patients with atopic dermatitis  Demographic information NR | Number and type: One multi-faceted and one single-component:   - Clinical interview form – Standard (a general diagnostic form with questions such as “have you ever been treated for this condition before?”) - **Pre-formed QPL** + Clinical interview form - Disease specific (an atopic dermatitis specific diagnostic form with questions such as “what is your trouble with atopic dermatitis) - Answer brochure (a multiple page brochure that has the same title as the QPL but provides answers to each question listed in the QPL, compiled by analyzing 52 physician responses to the questions on the QPL)   **Pre-formed QPL Description**  Recipients: Patients with atopic dermatitis  Personnel or setting: Outpatient dermatology clinic at city-based university hospital  Developers: This group, by compiling frequently asked questions from 41 dermatology clinics in university hospitals, and monitoring doctor-patient conversations for two years to see which questions were actually asked.  Purpose: To assist patients in bringing up questions they wanted to ask and contribute to better disease understanding.  Content: **Title** (“questions you may want to ask”); **Instructions for use** (3 sentences); **Questions** divided into sections including treatment in general, medicines, inheritance, daily life, and foods; **blank space** for additional questions with title “in addition” and instructions to use this space for additional questions  Format: Single printed page, white background, black text, 12-point font, title and **section headings** in bold, section titles presented on left slide of page with a bullet point and corresponding questions aligned on the right side of page using numbers. Instructions and the section for additional questions are centred at the top and bottom of page.  Delivery: researchers gave print QPL in clinic before appointment  Intensity/duration: 1 page, 5 sections, 17 questions  Copy of QPL: Figure 3 | QPL Use:   - 100% selected questions they wanted to ask doctors - All 17 questions were selected at least once   Content of Questions Asked:   - 63.5% of patients selected questions about good/bad foods - 53.8% selected questions about detergent and bath remedies - 50% selected questions about side effects   QPL Appraisal:   - 78% preferred intervention including QPL (vs. 21.2% preferring non QPL intervention) - 100% agreed that the QPL questions represented their concerns about atopic dermatitis - 27.3% found the QPL time-consuming to read, and two of these patients mentioned they were worried that if that if they gave QPL to doctors it would keep them from discussing issues other than those on the QPL |
| Glynne-Jones 2006 **(68)**  UK  Heterogeneous Cancer | To evaluate the impact of a QPL on patient question asking, information needs, active decision making, and patients’ perception of its usefulness. | Questionnaire | Sample size: 300 cancer patients, 29% prostate, 27% breast, 23% colorectal  Age: Median 67  Sex: 55% men  Education: NR | Number and type: One multi-faceted:   - Needs assessment (a multi-page questionnaire about patient information needs and satisfaction with the oncology clinic) + Pre-formed QPL (last page of needs assessment booklet)   **QPL Description**  Recipients: Patients with cancer attending weekly outpatient clinic  Personnel or setting: Outpatient oncology clinic in urban setting  Developers: Researchers from this group in collaboration with a professor from Royal Free Hospital School of Medicine, and with guideline recommendations (Commission for Health Improvement) and using a previously published QPL (from CancerBACUP)  Purpose: To enable patients to ask appropriate and relevant questions so they can obtain as much information as they require from each visit to the clinic  Content: **Title** “questions you may want to ask the doctor”; **Questions** divided into sections about “your condition”, “tests and treatment plan” and “other information/advice”; **blank space** after each section for additional questions  Format: Printed single page, included as last page of booklet with needs assessment questionnaire. White background, black text. Title in all capitals, larger font, and highlighted, page is divided horizontally into 3 sections, **each section headin**g is bolded and underlined in same font and size as questions, questions listed under section heading in bullet form, box with lines under each section/list of questions called “your questions”  Delivery: Patients were approached by a nurse after arriving at clinic. They filled out the questionnaire and received the QPL in the waiting room before consultation.  Intensity/duration: 1 page, 3 sections, 22 questions  Copy of QPL: Appendix 2 | QPL Use:  85% reported receiving QPL  QPL Appraisal (patients):   - 75% felt QPLs were helpful - 80% of these patients felt information received was “just right” - 20% felt they received insufficient information - 1 patient (of 159) felt they received far too much information - 33% were able to ask more questions about their disease because of QPL - 33% felt the doctor was busy and did not want to take up too much of their time - selected positive comments about QPL: provides good clear information, all questions answered accurately, information is to the point, able to get the truth which is what is wanted - Selected negative comments about QPL: junior doctors don’t have enough information, dissatisfied with information, notes are never to be found |
| Sciamanna 2006 **(69)**  US  Migraine | To assess the impact of a QPL on frequency and content of topics of discussion during consultation, patient satisfaction, and patient-clinician interactions, compared to usual care. | Randomized Controlled Trial | Sample size: 50 patients with migraines  Age: Mean 42  Sex: 86.5% female  Education: NR  Group differences:  Group 1: Instructed to access web-based QPL before consultation with provider  Group 2: Instructed to access web-based QPL after consultation | Number and type: One multi-faceted  **“**Myexpertdoctor” website intervention (same as (64), including: **Quiz** (patients had to answer 10-20 questions about their disease knowledge related to headache and its care) + **Plain language summary** (the website generates personalized feedback and information based on patient answers to the quiz) + **Pre-formed QPL** (Questions are suggested that pair with the personalized feedback)  **Pre-formed QPL Description**  Recipients: Patients with asthma  Personnel or setting: Subjects were identified through Blue Cross and Blue Shield health insurance memberships. No data provided on where patients consultations were (i.e., rural/urban, clinic/hospital)  Developers: This group, by reviewing evidence-based guidelines for suggested questions and then programming these questions into a website. They also selected and reviewed sites to include in the additional resources section. Funded by grants from the National Institutes of Health.  Purpose: To help patients ask the right questions, personalized to their particular condition and situation.  Content: **Title** “questions you should ask your doctor”; **Questions** personally tailored to patient needs which were assessed in initial quiz**; lay explanation** of why the patient should ask the physician each question, with justification related to their quiz answers; **Additional resources** a “more information” button that links to other websites for further reading and explanations of topics  Format: Web-based QPL. The website colours were shades of blue with black text and warnings in red (that arise from alarming answers to questions in the quiz section). Title and **section headings** in blue and larger font. Questions presented as a bullet form list at the top of webpage under title in 12-point font and blue colour because each question is a hyperlink that links to its corresponding section. Below the large question list is an individual section for each question, title in large blue font, with a lay explanation in black text for why the patient should ask that question, and a blue hyperlinked “more information” button in 12-point font.  Delivery: Patients were randomized to whether they used website including QPL before consultation or did not receive website. Time between receiving website and consultation NR.  Intensity/duration: Varies based on patient answers to quizzes. Based on examples provided could range from 0 – 6 questions, however maximum number of questions NR. All presented on one web-page where patient scrolls down to access more information (number of pages N/A). Each question and lay explanation presented as a new “section”  Copy of QPL: Details in online Appendix of (66). Provides link to website in article (myexpectdoctor.com) – unable to access. | QPL Use (within intervention group)   - 78.6% printed feedback (unclear if this includes QPL portion) - 28.6% brought printout to visit - 64.3% asked the questions suggested in QPL   Number of questions asked:   - Across groups, mean 5 topics discussed per consultation - Non-significant increase in number of questions asked in QPL group vs. control (5.5 vs. 4.3, p value NR)   Patient satisfaction with consultation: No group differences and generally (Mean 3.9 / 5) |
| Bolman 2005 **(70)**  The Netherlands  Coronary artery disease | To assess the impact of a QPL used in three consecutive visits following hospitalization for coronary artery disease compared to usual care. | Randomized Controlled Trial | Sample size: 118 patients with coronary artery disease  Age: Mean 61.9, SD 10.5  Sex: 19% female  Education: 49.6% less than high school, 28.2% secondary school, 22.2% university degree  Group differences:  Group 1: QPL  Group 2: Usual care | Number and type: One single-faceted:   - **Pre-formed QPL**   Recipients: Patients recently hospitalized for coronary artery disease attending follow up appointments  Personnel or setting: Outpatient cardiology clinic at a city-based university hospital  Developers: Previous research by this group (73) using a literature review on cardiology patient information needs, focus groups with cardiology patients, and expert review by experienced cardiologists  Purpose: To help patients prepare for the cardiologist visit, to structure patient-cardiologist information exchange, and to focus discussions around issues patients are most concerned about  Content: **instructions** explaining the purpose, procedure and practical use of the checklist**, questions** about nature of the disease, physical state, medication, risk factors and lifestyle, treatment, examinations, disease related family support and family issues, psychosocial problems, practical matters; **blank space for other questions**  Format: print  Delivery: Cardiologists were specifically asked by researchers to answer the questions asked by patients. Patients were mailed the QPL 1 week before their visit and were explicitly asked to fill in the QPL before their visit. They were also verbally instructed to bring the QPL to their visit and actively use it as a reminder during the visit. Patients were given QPL 3 times: before 1 month follow up, 4 month follow up and 10 month follow up appointments  Intensity/duration: 49 questions, 10 topics  Copy of QPL: Not provided. | QPL Use (within QPL group)   - 80% filled out QPL to prepare for first visit - Most common reason for not filling out QPL was they forgot - 73% used QPL during the visit - Most common reason for not using QPL was cardiologist did not ask for it, or questions were already answered - QPL use decreased at the second and third visit   QPL Appraisal:   - 80% would use QPL again at the next visit - 12% thought they had received more information using QPL than without it - 16% thought they had asked more questions than they would without QPL   Content of questions asked   - 46% of questions about disease related physical symptoms - 34% level of physical exertion or sports allowed - 31% question on how heart recovers - 31% how to distinguish symptoms from other minor health problems   Anxiety (Spielberger State Anxiety Inventory – score 20 [low anxiety] to 80 [high anxiety]: No significant group differences at any time points (p=0.08)  Information exchange (Perceived Information Adequacy questionnaire: No significant group differences at any time points  Patient participation in question asking: No significant group differences at any time points  Patient satisfaction with information exchange and cardiologist’s communicative attitude: No significant group differences at any time points  Disease knowledge (25 item questionnaire): Significant difference based on group and time (QPL group higher than control, 10 month follow up score higher than 1- and 4-month scores, p=0.02)  Consultation duration: significant difference based on group and time (control group longer than QPL, 4 month follow up longer than 1 month and 10 month follow up, p=0.004) |
| Butow 2004 **(71)**  Australia  Heterogeneous cancer | To evaluate the impact of a cancer consultation preparation package (including a QPL), compared to control booklet (informational booklet about hospital) | Randomized Controlled Trial | Sample size: 164 patients with cancer (29.5% breast)  Age: Mean 58.3  Sex: 54.4% female  Education: 38.45% year 10 or less, 23.75% year 12, 37.8% Postgraduate  Group differences:  Group 1: Cancer consultation preparation package  Group 2: Control booklet (information booklet about hospital) | Number and type: 1 multi-faceted:  Cancer consultation preparation package including: plain language summary (a booklet about decision making) + pre-formed QPL + informational brochure about patient legal rights + informational booklet about the hospital  **Pre-formed QPL Description**  Recipients: Patients with heterogeneous cancers attending first consultation  Personnel or setting: City-based teaching hospital outpatient clinic  Developers: Previously developed and evaluated by this group (72, 74)  Purpose: To improve and promote patient question asking as part of facilitating overall patient involvement in oncology consultations  Content: **Instructions** endorsing question asking and recommending that patients create a list of questions**; questions** suggested for inclusion; **blank space** for additional questions  Format: print booklet provided to patients by researcher at appointment before clinical consultation  Delivery: Intervention delivered at least 2 days before initial consultation with an oncologist  Intensity/duration: 19 questions  Copy of QPL: Not provided. Perhaps in previous literature listed in “developers” section | All data presented about intervention which includes QPL (no isolated QPL results)  Intervention use  98% used intervention  Content of questions asked   - The intervention group asked significantly more questions about prognosis than control (Median 1 vs. 0, p=0.001) - Intervention patients and caregivers asked significantly more new questions than control (Mean 12.9 vs. 9.3, p=0.15)   Number of questions asked: Intervention patients asked significantly more than control (Mean 13 vs. 9, p=0.009)  Patient active participation (initiates agenda, voices concerns, questions, interrupts etc.): No significant group differences in number of active behaviours (p=0.35)  Consultation duration: No significant differences, physicians spoke twice as long as patients, Mean 36 minutes  Patient Anxiety and depression: Significantly higher pre-consultation in intervention group (Mean 42 vs. 38, p=0.04). No significant group differences post-consultation, both decreased after consultation.  Patient Satisfaction with consultation and treatment decision: No significant group differences, both high  Physician satisfaction with decision making: no significant group differences  Physician patient-centred care behaviours: No significant differences (includes amount of information provided, encouraging patient participation, rapport-building behaviours, initiating social topics and issues, addressing patient anxiety, interrupting patients) |
| Bruera 2003 **(72)**  US  Breast Cancer | To compare the impact of a QPL compared to a general information sheet | Randomized Controlled Trial | Sample size: 60 patients with breast cancer  Age: Mean 53.75  Sex: NR  Education: 3% Less than high school, 32% High school, 65% college or higher  Group differences:  Group 1: QPL  Group 2: Plain language summary (control) | Number and type: Two single-component:   - Pre-formed QPL - Plain language summary (General information sheet about breast cancer)   **Pre-formed QPL Description**  Recipients: Patients with breast cancer  Personnel or setting: City based breast centre at a cancer centre  Developers: Current researchers by compiling questions and design elements from previously published QPLs and making changes based on this research group’s previous studies and clinical team expertise.  Purpose: NR  Content: **Instructions** to check the questions that you are interested in asking your doctor; **Questions** about breast cancer diagnosis, treatment, and prognosis; **blank space** for additional questions  Format: White background, black text, questions numbered on left hand side, presented one after the other with no space in between, 12-point font.  Delivery: Research nurse provided QPL to patients in an envelope in waiting room before consultation. No verbal instructions for use and no endorsement by the research nurse. Clinicians were not instructed to respond to the QPL.  Intensity/duration: 1 page, 22 questions  Copy of QPL: Appendix 2 | QPL appraisal (helpfulness, 0 to 10 rating):   - Overall helpfulness: QPL group significantly higher than control (8.5 vs. 6.2, p=0.005) - Helpfulness at communicating with physicians (QPL 7.9 vs. 5.7, p=0.01)   Satisfaction with consultation, doctor, and ability of doctor to answer patients’ questions: high in both groups, no significant differences  Number of questions asked: No significant group difference  Content of questions asked:  QPL group asked significantly more questions about diagnosis 2.5 vs. 1.4, p=0.025)  Patient and physician speaking time: no significant group differences in average patient and physician speaking times  Physician satisfaction with patient-clinician communication: No significant group differences  Consultation duration: in 67% of consultations, the physician estimated that consultation was no longer or shorter than expected, no significant group differences in this rating |
| Clayton 2003 **(73)**  Australia  Advanced Cancer (palliative care) | To develop a QPL and pilot test patients’ perception of QPL regarding usefulness, question asking behaviours, and overall feedback | Questionnaire | Sample size: 23 patients with advanced cancer  Age: 34.8% less than 60, 65.2% older than 60  Sex: 52.2% female  Education: 78.3% school certificate or below, 8.7% high school, 13% post-secondary | Number and type: One single component:   - **Pre-formed QPL**   Recipients: Palliative care patients and clinicians  Personnel or setting: 3 palliative care services in Sydney (at hospitals, palliative care units, and the community)  Developers: Current researchers by conducting focus groups and individual interviews with patients and clinicians. Interviews with patients explored the meaning of palliative care, questions patients had asked the palliative care service and questions they wished they had asked. Interviews with clinicians explored information thought to be most important to convey to patients during palliative care consultations, common questions asked by patients and their caregivers, and questions they felt would elicit useful information but patients and caregivers may have difficulty asking. All participants were asked for views on the draft of QPL and when it should be given.  Purpose: NR  Content: **table of contents; introduction** defining palliative care, endorsing question asking, explaining the purpose of the booklet; **instructions** suggesting that people may like to circle the questions they want to ask their doctor or nurse, suggesting that the booklet is kept for future reference and that patients may wish to use it with other members of the palliative care team, explaining that there may be some questions or topics that are not relevant to the patient or their stage of illness and suggesting that patients read the topic headings first and decide whether they want to read questions on that particular topic; **separate introductions** to caregiver and palliative care sections stating that these topics may not be relevant; **questions** separated into sections, with only one section printed on each page of the booklet; **blank spaces** after each section for additional questions;  Format: Printed booklet  Delivery: Given to patients in the waiting room before consultation by researcher. Patients had about 20 minutes to review QPL. Clinicians were specifically asked to endorse and refer to the QPL during consultation.  Intensity/duration: 9 sections, 114 questions  Copy of QPL: Not provided, detailed description in Appendix A | QPL appraisal: 70-100% found QPL to be helpful, useful, made it easier to ask questions, helped put questions into words, was easy to understand, and will be useful to me in the future  QPL length: 80% found QPL to be the right length  QPL Delivery: 80% had enough time to read QPL before consultation; 45% would have preferred to receive booklet earlier (2-3 days before consultation)  Anxiety:   - 88% decreased post consultation (State Trait Anxiety Inventory, median decrease 8 points) - 40% and <1% said there were questions that made me anxious, on day of consultation and 3 weeks after consultation   Physician QPL appraisal (n=23):   - 100% indicated QPL did not interfere with consultation flow - 87% QPL made it easier for patients to discuss sensitive issues - 100% (3/3 in pilot trial) would use it again - 100% (3/3 in pilot trial) found it useful |
| Brown 2001 **(74)**  Australia  Heterogeneous Cancer | To assess the impact of a QPL with physician endorsement, versus without physician endorsement, versus usual care. | Randomized Controlled Trial | Sample size: 318 patients with cancer (21% genitourinary, 19.5% breast, 17.3% gastrointestinal)  Age: Mean 56.12  Sex: 44.3% female  Education: 27% Less than 10 years, 41.5% high school, 9.4% post-secondary non university, 20.1% university, 1.9% unknown  Group differences:  Group 1: QPL with active endorsement from clinician  Group 2: QPL with no active endorsement from clinician  Group 3: Usual care (no QPL) | Number and type: One single-component and one multi-faceted:   - Pre-formed QPL - Pre-formed QPL + active endorsement from clinician   **Pre-formed QPL Description**  Recipients: patients with heterogeneous cancers, attending initial consultation with an oncologist  Personnel or setting: two city-based university teaching hospital outpatient cancer clinics  Developers: Previous research by this group (not in the manuscript)  Purpose: NR  Content: **title** “how to make the most of your time with the doctor”; **introduction** endorsing question asking as an activity useful to patients and welcomed by oncologist; **instructions** to circle questions they would like to ask and add additional questions; **questions** with **section heading “questions people often ask”**; **blank space** for additional questions.  Format: 12-point font, title and **section heading** bolded, questions in a numbered list  Delivery: Given to patients in waiting room by researcher with patients having at least 15 minutes to read and consider the QPL before consultation.  Intensity/duration: 17 questions, 4 lines for additional questions, 1 page, 1 section  Copy of QPL: Figure 1 | Number of Questions Asked:   - Median 9 questions per consultation - No significant difference in QPL + physician endorsement + QPL alone - No significant differences between both QPL groups vs. control - Patients with QPL asked more questions re: prognosis (p=0.039)   Content of questions asked: 47% (majority) about treatment  Consultation duration:   - QPL groups (analyzed together here) had significantly longer consultations (p=0.021) - mean duration 31 mins across groups - QPL + physician endorsement significantly shorter consultations vs. QPL alone and vs. control (28.5 mins vs. QPL alone 34.36 mins, vs. control 32.09 mins)   Patient satisfaction: no significant group differences  Patient anxiety (Spielberger State Anxiety Scale): Significantly higher in QPL alone vs. QPL + endorsement and vs. control (p=0.041)  Patient memory (recall of information at follow up phone call): Significantly higher in patients with QPL + endorsement, vs. QPL alone (p=0.036). Not significantly different from control.  Information needs (Information Needs Scale): No significant group differences |
| Martinali 2001 **(75)**  The Netherlands  Coronary Artery Disease | To investigate the impact of a QPL on patient- physician communication compared to an informational brochure. | Randomized Controlled Trial | Sample size: 103 patients with coronary artery disease  Age: Mean 64.3, SD 9.0  Sex: 19.4% female  Education: 45% less than high school, 25% high school, 29% college or university degree  Group differences:  Group 1: QPL + Plain language summary  Group 2: Plain language summary | Number and type: 1 multi-faceted and 1 single-component:   - Pre-formed QPL + plain language summary (informational brochure about coronary artery disease and related problems) - Plain language summary alone   **Pre-formed QPL Description**  Recipients: Patients attending consultation for coronary artery disease  Personnel or setting: Outpatient cardiology clinic at a city-based university hospital  Developers: Current researchers by compiling relevant questions from the published literature, and conducting two focus groups with patients. The draft was reviewed and adjusted by five experienced cardiologists and 15 patients with coronary artery disease.  Purpose: To help patients prepare for the cardiologist visit, to structure patient-cardiologist information exchange, and to focus discussions around issues patients are most concerned about  Content: **instructions** explaining the purpose, procedure and practical use of the checklist**, questions** about nature of the disease, physical state, medication, risk factors and lifestyle, treatment, examinations, disease related family support and family issues, psychosocial problems, practical matters; **blank space for other questions**  Format: print  Delivery: Cardiologists were specifically asked by researchers to answer the questions asked by patients. Patients were mailed the QPL 1 week before their visit, and were explicitly asked to fill in the QPL before their visit. They were also verbally instructed to bring the QPL to their visit and actively use it as a reminder during the visit.  Intensity/duration: 49 questions, 10 topics,  Copy of QPL: Not provided | QPL Use: 75% filled out QPL (otherwise forgot)  QPL Appraisal   - 100% found QPL useful - 96% found using QPL easy - 47% thought it did not help with information exchange - 71% would use it again   Content of questions asked: 60% marked questions about physical disease complaints, 45% psychosocial problems  Anxiety (Spielberger State Anxiety Inventory): Significantly higher in control group (Mean QPL 34.8 vs. control 39.7, p=0.02)  Patient participation: No significant group differences (Mean QPL 2.35 vs. control 2.21, p=0.50)  Information exchange (Perceived Information Adequacy): No significant group differences (Mean QPL 12.9 vs. control 12.5, p=0.73)  Patient satisfaction: No significant group differences (Mean QPL 23.9 vs. control 22.5, p=0.50)  Patient disease knowledge: No significant group differences (Mean QPL 41.5 vs. control 36.4, p=0.24)  Consultation duration: No significant group differences (Mean QPL 12 mins vs. control 10.3 mins, p=0.18) |
| Brown 1999 **(76)**  Australia  Heterogeneous cancer | To investigate the effects of a QPL endorsed and discussed by physicians alone or combined with a coaching intervention on question-asking behaviour, compared to usual care. | Randomized Controlled Trial | Sample size: 60 patients with cancer (27% breast, 12% lung, 12% prostate)  Age: Mean 53  Sex: 52% female  Education: 55% Less than 10 years, 10% High school, 10% tertiary non-university, 25% tertiary university  Group differences:  Group 1: QPL  Group 2: QPL + coaching  Group 3: Usual care | Number and type: One single-component, one multi-faceted:   - Pre-formed QPL alone - Pre-formed QPL + coaching (patient received QPL plus an interactive session with research psychologist covering: question generation – importance of asking questions, eliciting patient questions to compare and add to existing QPL; exploration of benefits of and barriers to question asking; rehearsal – imagine themselves in this situation and rehearse questions with researcher as a surrogate doctor)   **Pre-formed QPL Description**  Recipients: Patients with heterogeneous cancers visiting an oncologist for the first time  Personnel or setting: city-based tertiary referral teaching hospital  Developers: Current researchers by analyzing the content of 20 taped consultations, and consulting with two oncologists and two psychologists experienced in cancer research.  Purpose: NR  Content: **Title** “how to make the most of your time with the doctors”; **introduction; instructions for use; questions** about diagnosis, tests, treatment, prognosis, psychological issues, and support services available; **section heading** “questions people often ask”; **blank space** for questions  Format: title larger font than intro and questions and bolded, section heading italicized, questions numbered on left, white background and black text, 12-point font for questions and **section headings**.  Delivery: QPL was endorsed by the physician, and towards the end of consultation went through each category eliciting and answering questions according to a standard protocol.  Intensity/duration: 17 questions; 1 section; 4 lines for additional questions  Copy of QPL: Figure 2 | QPL Use:  Number of questions asked: No significant group difference (Median QPL 15, QPL + coaching 13, control 8.5); When intervention groups combined, QPL groups asked significantly more questions than control (p=0.043)  Content of questions asked: Combined QPL groups asked more questions about tests (Median 1 vs. 0, p =0.048)  Anxiety (Spielberger State Anxiety Scale): No significant group differences  Patient satisfaction with consultation: No significant group differences  Psychological adjustment to cancer (Mental Adjustment to Cancer scale): No significant group differences |
| Fleissig 1999 **(77)**  UK  Generic | To investigate the impact of a QPL on out-patients’ ability to prepare, prioritize, and remember questions to ask during their initial hospital consultation, compared to usual care. | Randomized controlled trial | Sample size: 1683 patients with dermatology (55%), gynaecology (29%), and orthopaedic (16%) appointments  Age: NR  Sex: 66% female  Education: NR  Group differences:  Group 1: QPL  Group 2: Usual care | Number and type: one single-component   - **Pre-formed QPL**   Recipients: New out-patients  Personnel or setting: city-based hospital outpatient clinic  Developers: Current researchers by modifying previous materials  Purpose: to help patients focus on what information they wanted and give patients “permission” to ask questions  Content: Page 1: **instructions** stating that doctor would be expecting patient to ask questions, printed questions were only suggestions, and advising patients to mark down priority of questions; Page 2: **title** “questions you may want to ask the doctor”**; questions** divided into sections: “your condition”, “tests and treatment plan”, “other information/advice”**; blank space** authors stated it could be used for additional questions but no instructions on QPL that the space is for that  Format: Two separate pages. Page 1 has instructions: formatted as a letter from hospital’s patient information program officer; Page 2 has the QPL: title in all capitals and bolded, **section headings** bolded, italicized and underlined, questions listed under headings in bulleted lists, page is landscape.  Delivery: Mailed to patients 2 weeks before consultation  Intensity/duration: 3 sections, 23 questions, 2 pages (one page was QPL and one separate page of instructions)  Copy of QPL: Figure 1 | QPL Use: 50% used QPL to help think of questions before consultation  Number of questions asked: 69% said all prepared questions were raised either by themselves or clinician  Patient Satisfaction with consultation: Patients who discussed all questions were significantly more satisfied than those who didn’t (p<0.001)  Common reasons QPL patients didn’t ask prepared questions: questions already answered, forgot, not enough time, didn’t want to waste doctors time, questions not important, doctor seemed to dislike questions, too embarrassing, no right moment |
| Butow 1994 **(78)**  Australia  Heterogeneous cancer | To assess the impact of a QPL compared to an informational sheet | Randomized Controlled Trial | Sample size: 142 patients with cancer (43% breast)  Age: Mean 51 years  Sex: 84% female  Education: NR  Group differences:  Group 1: QPL  Group 2: Control (Informational sheet about services available through the regional Cancer Council) | Number and type: 1 single-component:   - Pre-formed QPL   **Pre-formed QPL Description**  Recipients: Patients with heterogeneous cancer attending their first oncology consultation  Personnel or setting: Suburban cancer center outpatient clinic at a university teaching hospital  Developers: Previous research by this group (not in the manuscript).  Purpose: To encourage patient question-asking and participation in consultation  Content: **title** “how to make the most of your time with the doctor”; **introduction; instructions; questions** with subtitle “questions people often ask”; **blank lines** for additional questions with subheading “your questions today”; **importance rating** of additional questions (rate importance 1 – 5)  Format: titles and subsection headings bolded, text not bolded, questions and blank lines for additional questions numbered on left margin, white background, black text  Delivery: Given to patients in the waiting room by researcher before consultation and with at least 10 minutes for patients to review QPL  Intensity/duration: 11 questions, 1 page, 10 blank lines for additional questions, labelled 1 – 5 (2 lines per question)  Copy of QPL: Figure 1 | Content of questions asked: Significantly more questions asked about prognosis in QPL group compared to control (35% vs. 16%, p=0.03)  Number of questions asked: No significant group differences  Patient talk time: no significant group differences  Predictors of question asking: People aged <51 (p<0.001), females (p<0.001), and outpatients (p<0.05) asked significantly more questions than people older than 51, males, and inpatients  Satisfaction with consultation: No significant group differences, both groups high  Psychological adjustment to cancer: No significant group differences  Patient recall of information: no significant group differences |
| Thompson 1990 **(79)**, study 2  US  Obstetric and gynecological patients | To assess patients’ perception of physician attitudes towards question asking compared to pre-formed questions | Randomized Controlled Trial | Sample size: 49 obstetric and gynecological patients  Age: Mean 30  Sex: 100% women  Education: NR  Group differences:  Group 1: Pre-formed QPL  Group 2: Patient-generated QPL  Group 3: Control (Questionnaire about waiting room) | Number and type: Two single-component   - Pre-formed QPL - Patient generated QPL   **QPL Descriptions**  Recipients: *Both:* obstetrician/gynecologist patients  Personnel or setting: *Both:* 2 outpatient obstetrician/gynecology clinics  Developers: *Both:* NR  Purpose: *Both:* NR  Content: *Pre-formed QPL*: **list of common questions and topics** about diagnosis, cause of problem, name of medication, side effects of medication, and when to return for checkup; *Patient-generated QPL:* **introduction and instructions** written from the physician, endorsing question asking, communication and cooperation and stating to “please feel free to ask questions in the office visit today” (no blank space to write questions)  Format: *Both:* print  Delivery: *Both:* Given to patients by the receptionist before consultation  Intensity/duration: *Pre-formed QPL*: 13 items; *Patient-generated QPL:* NR  Copy of QPL: Not provided | No significant differences between pre-formed vs. patient generated QPLs for any measure. All results below combined the two QPLs vs. control  Anxiety (State Anxiety subscale): No significant difference between QPL groups and control  Patient satisfaction with consultation: Combined QPL groups significantly higher than control (p<0.05)  Patient satisfaction with information: Combined QPL groups significantly higher than control (p<0.05)  Number of questions asked: No significant difference between QPL groups vs. control |
| Tabak 1988 **(80)**  US  Generic | To pilot test the impact of a QPL on information seeking skills, question asking, and satisfaction with car, compared to an informational booklet. | Randomized Controlled Trial | Sample size: 67 patients of family physicians (diagnosis NR)  Age: Mean 35  Sex: 80.6% female  Education: Mean 13.06 years of education  Group differences:  Group 1: QPL  Group 2: Control (Informational booklet about clinic hours and services) | Number and type: One single-faceted:   - **Pre-formed QPL**   Recipients: Patients at a family medicine clinic  Personnel or setting: City-based family medicine clinic associated with university department of medicine.  Developers: Created by current group using results of previous research that content-analyzed research interviews with patients (not in manuscript)  Purpose: To encourage patients to ask questions, legitimize active information seeking as part of the patient role and help patients recognize and find words for their requests.  Content: **introduction:** education about importance of recognizing information needs and encouraging patients to verbalize requests for information; **instructions:** written at 7^th^ grade reading level; **questions** about physical symptoms, reasons for symptoms, treatment, reasons for treatment, and impact on life. Written at 2^nd^ grade reading level; **blank page** at the back of QPL booklet with space for additional questions  Format: Printed booklet  Delivery: Provided to patients by researcher before appointment. Patients were given as much time as they needed with QPL and were specifically asked if they were finished with QPL before medical student accompanied them to consultation.  Intensity/duration: 33 questions  Copy of QPL: Not provided | Number of questions asked: No significant group difference (Mean QPL 7.46 vs. control 5.63) |
